# Supplementary material for: Early use of Antibiotics for at Risk CHildren with InfluEnza (ARCHIE): protocol for a double-blind, randomised, placebo-controlled trial
Source: BMJ Open. 2018 May 16;8(5):e021144. doi: 10.1136/bmjopen-2017-021144 (PMC5961556; doi:10.1136/bmjopen-2017-021144)
Supplement: Supplementary data [file bmjopen-2017-021144supp001.pdf]

Appendix A: Laboratory analysis of throat swabs

Where possible, throat swabs will be obtained from participants at baseline for culture and sensitivity, and in a sub group at 3, 6 and 12 months. The swab will have a broth medium to improve retrieval of the target organisms, which are *Staphylococcus aureus*, *Streptococcus pneumoniae* and *Haemophilus influenzae*. Aliquots of the broth will be pipetted onto selective agar for each of the different organisms. A fourth plate, a selective agar for *Haemophilus influenzae* with ampicillin in the medium at 2mg/L will also be inoculated. Identification of target organisms will be performed in line with Public Health England Standards for Microbiological Investigation methods and susceptibility testing performed in accordance with the latest British Society of Antimicrobial Chemotherapy (BSAC) guidelines. The table below summarises the antimicrobials that will be used for susceptibility testing. Residual broth and isolates of the target organisms will then be put into long-term storage for potential further molecular analysis of resistance and determination of phenotypic resistance. The broth will be frozen to either -70°C or -80°C, in effect snap frozen. Thus, there will be no preservation of intact cells. Assessment of minimum inhibitory concentrations (MICs) will be performed using the agar stipulated in BSAC guidelines.

**Antimicrobial**	**Method**	***S.aureus***	***S.pneumoniae***	***H.influenzae***
Penicillin	MIC	No	Yes	No
Amoxicillin	MIC	No	No	Yes
Co-amoxiclav	MIC	Yes	Yes	Yes
Cefoxitin	MIC	Yes	No	No
Cefotaxime	MIC	No	Yes	Yes
Moxifloxacin	MIC	Yes	Yes	Yes
Erythromycin	MIC	Yes	Yes	No
Nalidixic acid	Disc 30µg	No	No	Yes

Table footnote:

MIC = Minimum Inhibitory Concentration
S. aureus = Staphylococcus aureus
S. pneumoniae = Streptococcus pneumoniae
H. influenzae = Haemophilus influenzae

Appendix B: Data collection

Procedures	Enrolment		Post allocation (trial)			Close out
	V1	V2	Diary	T1	T2	NR
Eligibility assessment	x					
Informed consent +/- assent		x				
Baseline assessment		x				
Nasal swab		x				
Throat swab*		x				
Randomisation		x				
Dispensing of study drug		x				
Allocation of study diary and pack		x				
Adverse events assessment				x	x	
Medical notes review						x
**Assessments**						
Age		x				
Sex		x				
Co-morbidity		x				x
Household smoking status		x				
Vaccination status		x				x
Antivirals/other medications		x				x
Regular medications						x
Heart rate		x				
Respiratory rate		x				
Baseline annual consultation rate						x
Re-consultations due to clinical deterioration						x
Duration of fever		x	x	x	x	
Duration of symptoms		x	x	x	x	
Further medications and/or further investigations						x
Adverse events				x	x	x
Hospitalisations/death				x	x	x
Health care resource use			x			x
EQ-5D-Y proxy		x	x			

V1 = Visit 1; V2 = Visit 2 (conducted within 24 hours of visit 1); T1 = telephone follow-up 1 (one week after randomisation); T2 = telephone follow-up 2 (two weeks after randomisation); NR = Notes Review (i.e. review of participant’s medical record).
