# Supplementary material for: Early use of Antibiotics for at Risk CHildren with InfluEnza (ARCHIE): protocol for a double-blind, randomised, placebo-controlled trial
Source: BMJ Open. 2018 May 16;8(5):e021144. doi: 10.1136/bmjopen-2017-021144 (PMC5961556; doi:10.1136/bmjopen-2017-021144)
Supplement: Author's manuscript [file bmjopen-2017-021144.draft_revisions.pdf]

BMJ Open is committed to open peer review. As part of this commitment we make the peer review history of every article we publish publicly available.

When an article is published we post the peer reviewers' comments and the authors' responses online. We also post the versions of the paper that were used during peer review. These are the versions that the peer review comments apply to.

The versions of the paper that follow are the versions that were submitted during the peer review process. They are not the versions of record or the final published versions. They should not be cited or distributed as the published version of this manuscript.

BMJ Open is an open access journal and the full, final, typeset and author-corrected version of record of the manuscript is available on our site with no access controls, subscription charges or pay-per-view fees (http://bmjopen.bmj.com).

If you have any questions on BMJ Open's open peer review process please email info.bmjopen@bmj.com

BMJ Open

The early use of Antibiotics for at Risk CHildren with InfluEnza (ARCHIE): protocol for a double-blind randomised placebo-controlled trial

Journal:	*BMJ Open*
Manuscript ID	bmjopen-2017-021144
Article Type:	Protocol
Date Submitted by the Author:	13-Dec-2017

Complete List of Authors:	Wang, Kay; Nuffield Department of Primary Care Health Sciences<br>Carver, Tricia; University of Oxford, Nuffield Department of Primary Care Health Sciences<br>Tonner, Sharon; University of Oxford, Nuffield Department of Primary Care Health Sciences<br>Semple, Malcolm G; University of Liverpool, Women's and Children's Health, Institute of Translational Medicine<br>Hay, Alastair; University of Bristol, Centre for Academic Primary Care<br>Moore, Michael; University of Southampton Medical School, Primary Care Medical Group<br>Little, Paul; University of Southampton, Primary Care and Population Science<br>Butler, C; University of Oxford, Nuffield Department of Primary Care Health Sciences<br>Farmer, Andrew; University of Oxford, Nuffield Department of Primary Care Health Sciences<br>Perera, Rafael; University of Oxford, Nuffield Department of Primary Care Health Sciences<br>Yu, Ly-Mee; University of Oxford, Nuffield Department of Primary Care Health Sciences<br>Mallett, Susan; University of Birmingham, School of Health & Population Sciences<br>Wolstenholme, Jane; University of Oxford, Nuffield Department of Population Health<br>Harnden, Anthony; Oxford University, Nuffield Department of Primary Care Health Sciences

Keywords:	Paediatric infectious disease & immunisation < PAEDIATRICS, Respiratory infections < THORACIC MEDICINE, Clinical trials < THERAPEUTICS

SCHOLARONE™
Manuscripts

The early use of Antibiotics for at Risk CHildren with InfluEnza (ARCHIE): protocol for a double-blind randomised placebo-controlled trial

Kay Wang[1], Tricia Carver[1], Sharon Tonner[1], Malcolm G Semple[2], Alastair D Hay[3], Michael Moore[4], Paul Little[4], Christopher Butler[1], Andrew Farmer[1], Rafael Perera[1], Ly-Mee Yu[1], Susan Mallett[5], Jane Wolstenholme[6], Anthony Harnden[1]

[1]Nuffield Department of Primary Care Health Sciences, University of Oxford, Radcliffe Primary Care, Woodstock Road, Oxford OX2 6GG, UK; [2]Women's and Children's Health, Institute of Translational Medicine, University of Liverpool, Alder Hey Children's Hospital, Eaton Road, Liverpool L12 2AP, UK; [3]University of Bristol, Centre for Academic Primary Care, Canynge Hall, 39 Whatley Road, Bristol BS8 2PS, UK; [4]Academic Unit: Primary Care and Population Sciences, University of Southampton, Aldermoor Health Centre, Aldermoor Close, Southampton SO16 5ST, UK; [5]Institute of Applied Health Research, University of Birmingham, Edgbaston, Birmingham B15 2TT, UK; [6]Health Economics Research Centre, Nuffield Department of Population Health, University of Oxford, Richard Doll Building, Old Road Campus, Oxford OX3 7LF, UK.

Kay Wang, NIHR Postdoctoral Fellow; Tricia Carver, Senior Trial Manager; Sharon Tonner, Trial Manager; Malcolm G Semple, Senior Lecturer in Child Health; Alastair Hay, Professor of Primary Care; Michael Moore, Professor in Primary Health Care Research; Paul Little, Professor of Primary Care Research; Christopher Butler, Professor of Primary Care; Andrew Farmer, Professor of General Practice; Rafael Perera, Professor of Medical Statistics; Ly-Mee Yu, Associate Professor; Susan Mallett, Senior Lecturer in Medical Statistics; Jane Wolstenholme, Associate Professor of Health Economics; Anthony Harnden, Professor of Primary Care.

Correspondence to: Kay Wang, kay.wang@phc.ox.ac.uk

For peer review only

For peer review only - http://bmjopen.bmj.com/site/about/guidelines.xhtml

ABSTRACT

Introduction. Influenza and influenza-like illness (ILI) create considerable burden on health care resources each winter. Children with pre-existing conditions such as asthma, diabetes mellitus, and cerebral palsy are amongst those at greatest risk of clinical deterioration from influenza/ILI. The ARCHIE trial aims to determine whether early oral treatment with the antibiotic co-amoxiclav reduces the likelihood of re-consultation due to clinical deterioration in these 'at risk' children.

Methods and Analysis: The ARCHIE trial is a double-blind parallel randomised placebo-controlled trial. 'At risk' children aged 6 months to 12 years inclusive who present within the first five days of an ILI episode will be randomised to receive a five-day course of oral co-amoxiclav 400/57 twice daily or placebo. Randomisation will use a non-deterministic minimisation algorithm to balance age and seasonal influenza vaccination status.

To detect respiratory virus infections, a nasal swab will be obtained from each participant before commencing study medication. To identify carriage of potential bacterial respiratory pathogens, we will also obtain a throat swab where possible.

The primary outcome is re-consultation in any health care setting due to clinical deterioration within 28 days of randomisation. We will analyse this outcome using log-binomial regression model adjusted for region, age and seasonal influenza vaccination status.

Secondary outcomes include duration of fever, duration of symptoms, and adverse events. Continuous outcomes will be compared using regression analysis (or equivalent nonparametric method for non-normal data) adjusting for minimisation variables. Binary outcomes will be compared using Chi-squared/Fisher's exact test and log-binomial regression.

Ethics: The ARCHIE trial has been reviewed and approved by the North West-Liverpool East Research Ethics Committee, Health Research Authority, and Medicines and Healthcare Products Regulatory Agency. Our findings will be published in peer-reviewed journals and disseminated via our study website (www.archiestudy.com) and links with relevant charities.

Trial Registration: ISRCTN 70714783; EudraCT: 2013-002822-21

For peer review only - http://bmjopen.bmj.com/site/about/guidelines.xhtml

STRENGTHS AND LIMITATIONS OF THIS STUDY

- This randomised controlled trial aims to determine whether early treatment with the antibiotic co-amoxiclav reduces the likelihood of re-consultation due to clinical deterioration in children with influenza/influenza-like illness (ILI) who have known risk factors for influenza-related complications.
- The study will also assess cost-effectiveness and potential impact on long-term antibiotic resistance of early co-amoxiclav use in 'at risk' children.
- Recruitment will take place in England and Wales across a range of primary and ambulatory health care settings where 'at risk' children may potentially present with influenza/ILI.
- It will not be possible to determine whether children with ILI have influenza or other types of infection at the point of randomisation.
- Expansion of the UK childhood seasonal influenza vaccination programme may adversely impact upon recruitment.

For peer review only

For peer review only - http://bmjopen.bmj.com/site/about/guidelines.xhtml

INTRODUCTION

Background and rationale

Influenza is a viral infection which circulates mainly during winter and is a well-recognised risk factor for bacterial complications. 'At risk' children are more prone to becoming seriously unwell from influenza-related complications than otherwise healthy children.

'At risk' children are defined as children with underlying medical conditions or risk factors associated with an increased likelihood of developing complications from influenza/influenza-like illness (ILI). Based on guidance from the UK Department of Health(1) and the US Advisory Committee on Immunization Practices (ACIP),(2) 'at risk' groups include patients with chronic respiratory, cardiac, renal, liver and neurological conditions, as well as diabetes mellitus and immunosuppression. There is also systematic review evidence, mainly based on data from children under 2 years of age, that premature birth (i.e. delivery before 37 weeks gestation) is a risk factor for influenza-related complications.(3)

Based on data from Hospital Episode Statistics and the Office for National Statistics, 490,000 GP consultations and 4200 hospitalisations due to seasonal influenza occur each year in children aged 14 years or younger.(4) This results in a cost to the NHS of approximately £6.7 million due to hospitalisations(5) and £18 million due to primary care consultations.(6) The overall NHS and wider socioeconomic burden is likely to be greater due to additional costs incurred by critical care admissions, Accident and Emergency Department attendances, clinical interventions (investigations and medications) and parental productivity losses (days off work and childcare costs).

Based on recommendations from the Joint Committee on Vaccination and Immunisation,(7) the UK introduced universal seasonal influenza vaccination for all children aged 2 and 3 years from 2013/14, extending to all children aged 4 years during the 2014/15 influenza season. In 2017/18, Public Health England also initiated vaccination of children aged four to five years at school rather than in primary care, and included children in year 4 of school (aged eight to nine years) in the childhood influenza vaccination programme.(8)

Vaccine uptake of 55.7% has been observed in areas using school delivery models, compared to only 34.7% when general practice delivery models are used.(9) However, vaccination rates in children with 'at risk' conditions have not improved from around 40% since 2013/14.(10) Furthermore, effectiveness of the UK 2015/16 seasonal influenza vaccination was only around 58% in children aged 2 to 17 years.(11) Evidence for treatment with neuraminidase inhibitors in 'at risk' children is also weak. Oseltamivir has not been demonstrated to provide significant benefit in terms of shortening symptom duration(12) or reducing acute exacerbations(13) in children with asthma.

Although there is a substantial evidence base underpinning recommendations that routine antibiotic treatment is not indicated for viral respiratory tract infections,(14) there is also preliminary evidence to suggest that early antibiotic use may be beneficial in preventing clinical deterioration and complications due to influenza. The results of a small randomised placebo-controlled trial suggest that early treatment with the antibiotic sultamicillin in children presenting with influenza/ILI significantly reduces the incidence of pneumonia.(15) Additionally, observational data from school aged children presenting in primary care with cough and fever found that duration of fever was

For peer review only - http://bmjopen.bmj.com/site/about/guidelines.xhtml

significantly shorter in children with laboratory-confirmed influenza who received antibiotics (mostly amoxicillin) at an early stage during their illness.(16)

In recognition of the potentially serious clinical and socioeconomic consequences of bacterial complications of influenza, the UK government stockpiles the antibiotic co-amoxiclav for use during influenza epidemics and pandemics. Co-amoxiclav is the antibiotic of choice due to its coverage of both *Streptococcus pneumoniae* and *Staphylococcus aureus*, the most commonly observed bacterial co-infections in patients with laboratory-confirmed influenza.(17) In particular, *Staphylococcus aureus* co-infections are associated with worse clinical outcomes in patients with laboratory-confirmed influenza(18) and were found in nearly half of influenza-related deaths between October 2004 and September 2012 among US children from whom at least one bacterial pathogen was identified from a normally sterile site.(19)

At the same time, antibiotic prescribing is a major driver of antimicrobial resistance,(20) which is well recognised as an emerging threat to the treatment of serious bacterial infections. Data on the potential impact of early co-amoxiclav use on development and duration of antimicrobial resistance are therefore needed to guide future national guidance on supply and use of this antibiotic during periods of high influenza activity. A clear, evidence-based understanding of the benefits versus harms will in turn support local antibiotic stewardship programmes and better informed, more appropriate decisions about antibiotic prescribing and use in the community.(21)

METHODS AND ANALYSIS

Objectives

Our primary objective is to determine whether early treatment with co-amoxiclav reduces the likelihood of re-consultation due to clinical deterioration in 'at risk' children with influenza/influenza-like illness (ILI).

Other objectives are:

- To determine whether early treatment with co-amoxiclav reduces duration of fever in 'at risk' children with influenza/ILI.
- To determine whether early treatment with co-amoxiclav reduces duration of symptoms in 'at risk' children with influenza/ILI.
- To compare further intervention rates in 'at risk' children with influenza/ILI treated with co-amoxiclav versus placebo.
- To compare adverse events in 'at risk' children with influenza/ILI treated with co-amoxiclav versus placebo.
- To assess the cost, outcomes and cost-effectiveness of early treatment with co-amoxiclav in 'at risk' children with influenza/ILI versus placebo.
- To determine the impact on long-term respiratory bacterial carriage and antibiotic resistance of early treatment with co-amoxiclav versus placebo in 'at risk' children with influenza/ILI.

Study design

For peer review only - http://bmjopen.bmj.com/site/about/guidelines.xhtml

The ARCHIE study is a multi-centre double-blind randomised placebo-controlled trial. 'At risk' children (i.e. children with known risk factors for influenza-related complications) who present within the first five days of an influenza-like illness and who are not considered by their clinician to require immediate antibiotic treatment or hospitalisation, will be randomised to receive a 5-day course of co-amoxiclav 400/57 mg or a matching placebo. Nasal swabs will be obtained from all participants to detect influenza infection. Throat swabs for culture and sensitivity will also be obtained at baseline where possible. The primary outcome is re-consultation due to clinical deterioration within 28 days of randomisation.

Study participants

We will recruit 'at risk' children aged six months to 12 years inclusive who present within the first five days of an influenza-like illness. Box 1 summarises our definition of 'at risk' groups. Rather than being an exhaustive list, this definition is intended to guide clinicians in identifying which children are likely to be at greater risk of influenza-related clinical deterioration or complications. Health care professionals are also advised to use their clinical judgement to identify 'at risk' children. Children with other potential risk factors who may be suitable to take part may be discussed with a medically qualified member of the research team. Children who require immediate antibiotics or hospital admission for treatment of an influenza-related complication (based on their clinician's judgement) will not be eligible to take part. Box 2 summarises our full inclusion and exclusion criteria.

BOX 1: 'AT RISK' GROUPS

Respiratory

- Asthma requiring continuous or repeated use of controller therapy (e.g. inhaled steroids, leukotriene receptor antagonists, long-acting beta agonists, systemic steroids).
- Admitted to hospital with exacerbation of asthma within the last 12 months.
- Admitted to hospital with bronchiolitis or pneumonia within the last 12 months.
- Recurrent viral wheeze (3 or more episodes within the last 12 months).
- Bronchopulmonary dysplasia.

Cardiac

- Congenital heart disease being actively managed or monitored by cardiology team.
- Chronic heart failure being actively managed or monitored by cardiology team.

Neurological

- Chronic neurological or neuromuscular disorder which compromises respiratory function (e.g. cerebral palsy).

Renal

- Chronic kidney disease defined as either of the following:
- Impaired eGFR[§] (estimated glomerular filtration rate) measurement within the last 12 months.
- Known hereditary or structural kidney abnormality with or without impairment in eGFR.
- Nephrotic syndrome.
- Kidney transplantation.

Liver[§§]

For peer review only - http://bmjopen.bmj.com/site/about/guidelines.xhtml

- Cirrhosis
- Biliary atresia
- Chronic hepatitis

Immunodeficiency

- Asplenia or splenic dysfunction.
- HIV infection.
- Undergoing chemotherapy leading to immunosuppression.
- Taking systemic steroids at a dose equivalent to prednisolone 20mg or more per day (any age) or >=1mg per kg per day (children under 20kg).

Other

- Diabetes mellitus (type 1 or type 2) or other metabolic condition.
- Genetic abnormality (e.g. Down's syndrome)
- Sickle cell disease
- Malignancy
- Prematurity (born before 37 weeks gestation) in children aged 6 to 23 months.

§Impaired eGFR is defined as an eGFR measurement of 59 ml/min/1.73m^2 or less within the last 12 months before study entry. However, to enter the trial the following two conditions must also be satisfied:

1) eGFR >=30 ml/min/1.73m^2 based on most recent measurement within the last 12 months;
2) no reason to suspect further deterioration in eGFR at time of study entry.

§§Children with mild or moderate liver disease may enter the trial. However, to minimise the risk of serious hepatic complications related to study medication, children with severe liver disease may not enter the trial. Severe liver disease is defined as hepatic impairment associated with any of the following: jaundice, impaired coagulation/increased bleeding risk, bilirubin persistently greater than 50 micromol/litre (two measurements within last 12 months).

BOX 2: Study inclusion and exclusion criteria

Inclusion Criteria

- Aged 6 months to 12 years inclusive.
- In 'at risk' category.
- Presenting with influenza-like illness (i.e. cough and fever*) during influenza season.
- Presenting within 5 days of symptom onset.
- Permanently registered at a general practice in UK.
- Parent /guardian able to complete study diary and questionnaires.

Exclusion Criteria

The participant may not enter the trial if ANY of the following apply:

For peer review only - http://bmjopen.bmj.com/site/about/guidelines.xhtml

- Known contraindication to co-amoxiclav.
- Child given antibiotics for treatment of an acute infection within the last 72 hours.
- Child requires immediate antibiotics (clinician's judgement).
- Child requires immediate hospital admission for treatment of an influenza-related complication (clinician's judgement).
- Child has been observed on hospital ward or ambulatory care unit for longer than 24 hours.
- Presence of any reason to prevent healthcare professional from obtaining nasal swab.
- Child with known cystic fibrosis.
- Child previously entered into the ARCHIE study.
- Child has been involved in another medicinal trial within the last 90 days.

*Fever will be defined as any of the following: child-reported fever, parent-reported fever or temperature >37.8°C (axillary or tympanic temperature measurement).

Recruitment

We will recruit study participants from a range of health care settings where 'at risk' children from the community present with influenza/ILI. Recruiting sites will include general practices, walk-in centres and hospitals. Identification of participants at these sites may be supported by the use of participant identification centres. Given the seasonal nature with which influenza circulates in the community, each recruitment season will commence at the beginning of October and continue until the end of March the following year, or later if influenza continues to circulate above seasonal thresholds.

Before each recruitment season, we will ask participating general practices to perform database searches to identify children within our target age range who are in 'at risk' groups. A medically qualified individual will manually screen the results of the database search to exclude any children who would not be suitable to invite to take part in the study. Recruiting sites may also install a study reminder screen prompt, which will appear when the electronic medical records of potentially suitable children are accessed. Participating hospitals will also be asked to identify potential recruits through specialist outpatient clinics which manage children with 'at risk' conditions.

To further raise awareness about the trial and opportunities for participation, we will provide recruiting sites with study promotional materials including posters and short information leaflets, which summarise our study aims and direct families of potential participants to our study website (www.archiestudy.com). We will also liaise with recruiting sites to raise awareness via local news outlets, social media, and charities representing relevant patient groups. Parents who express an interest in allowing their child to take part in the study will be able to contact the study team or look at the study website to find their nearest recruiting site.

During each recruitment season, children who present at recruiting sites with influenza/ILI will be screened to determine whether they are eligible to take part in the trial. Suitably trained health care professionals will then obtain informed consent for study participation from the child's parent or guardian and complete study enrolment and randomisation procedures either at the recruiting site or at the child's home within 24 hours of confirming their eligibility for study participation.

For peer review only - http://bmjopen.bmj.com/site/about/guidelines.xhtml

Intervention

Participants will be randomised to receive either co-amoxiclav 400/57 (amoxicillin 400 mg as trihydrate and clavulanic acid 57 mg as potassium salt/5 mL when reconstituted with water) or a matching placebo, which will be taken orally twice daily for 5 days. Study medication doses based on age, as well as weight in participants aged 6 to 23 months, will be calculated according to the standard dosing regimen for co-amoxiclav 400/57 recommended by the British National Formulary (BNF, see box 3). Study medication will be provided as a dry powder, which health care professionals will reconstitute with water once the participant has been randomised.

Medically qualified individuals will use their clinical judgement when advising on study medication doses for any children to whom they feel that standard BNF dosing recommendations should not apply.

For peer review only

BOX 3: Study medication dosing regimen

**Child's age**	**Study medication dose**
**6 months to 23 months**	
Under 6 kg	Calculate dose according to British National Formulary instructions for co-amoxiclav 400/57. Advise two doses daily for 5 days.
6.0 – 7.9 kg	1 ml twice daily for 5 days
8.0 – 10.9 kg	1.5 ml twice daily for 5 days
11.0 – 12.9 kg	2 ml twice daily for 5 days
**2 to 6 years**	2.5 ml twice daily for 5 days
**7 to 12 years**	5 ml twice daily for 5 days

Baseline study procedures

Health care professionals will record baseline data from study participants on age, sex, co-morbidities, household smoking status, influenza vaccination status, medications given during the current illness episode, duration of fever, and duration of symptoms. Heart rate and respiratory rate will also be measured and recorded at baseline. Other baseline characteristics (medical conditions, regular medications, vaccinations, and acute consultations which occurred up to 12 months before randomisation) will be extracted from participants' medical records.

A nasal swab will be obtained from each participant at baseline. Nasal swabs will be placed in viral transport medium and sent by post to Alder Hey Children's Hospital microbiology department for analysis, where they will be analysed by real-time Polymerase Chain Reaction (PCR) analysis to detect influenza and distinguish influenza A, B and A/H1N1 2009 pandemic subtypes. Residual medium will be retained for potential future detection of other pathogens. Where possible, a throat

For peer review only - http://bmjopen.bmj.com/site/about/guidelines.xhtml

swab will also be obtained from the participant for culture and sensitivity. In a subgroup, further throat swabs will be collected after three, six and twelve months. Appendix A provides further details of the laboratory analyses which will be performed on throat swab samples.

Outcomes

The primary outcome is re-consultation due to clinical deterioration within 28 days of randomisation. Clinical deterioration is defined as any of: worsening symptoms, development of new symptoms or development of a complication requiring medication or hospitalisation. This definition is based on that used by the GRACE (Genomics to combat Resistance against Antibiotics in Community-acquired lower respiratory tract infection in Europe) consortium in to define re-consultations due to clinical deterioration in patients with lower respiratory tract infections.(22)

Secondary outcomes include duration of fever and duration of symptoms from time of randomisation, and medication prescriptions and/or further investigations, adverse events, hospitalisations or deaths, all within 28 days of randomisation. For the purpose of the economic evaluation, outcomes include healthcare resource utilisation and parental/informal care costs within 28 days from randomisation and health related quality of life as measured by the EQ-5D-Y proxy at baseline and days 4, 7, 14 and 28.

Data collection

Appendix B presents a complete summary of data collected during the trial. Data obtained at the baseline assessment, week 1 and week 2 follow-ups, as well as data on safety events, will be entered on paper case report forms before being sent to the main trial office for entry using OpenClinica open source software, version 3.13 Waltham MA, USA, a validated online electronic data capture system. All data will be double entered to ensure accuracy. Data extracted from the medical notes of children recruited from general practices will be directly transcribed into OpenClinica at the child's general practice. The main trial office will contact the general practices of children recruited from hospitals or walk-in centres to request relevant data from their medical notes, which will then be entered by a member of the trial team.

A parent or guardian will be asked to complete and return by post a series of four one-week study diaries on behalf of the participant to provide data on doses of study medication given to the child, axillary temperature, symptoms, adverse events, daily activities and childcare, child's health-related quality of life measured by the EQ-5D-Y proxy and health service contacts such as hospitalisations and visits to the GP. Health care professionals will also contact each participant's parent or guardian by telephone one week and two weeks after randomisation to collect data on duration of fever and adverse events. Primary outcome data will be extracted from participants' medical notes at least three months after randomisation. This is to allow records of any hospital admissions or consultations in non-general practice settings to be captured in the child's general practice medical record. Data on duration of symptoms will be collected from study diaries completed by participants' parents/guardians. Data on duration of fever will also be collected from study diaries and supplemented by follow-up telephone calls with parents/guardians one week and two weeks after randomisation. During these telephone calls, parents/guardians will be asked whether or not their child's fever has settled and if so, when. Data on adverse events will also be collected at these

For peer review only - http://bmjopen.bmj.com/site/about/guidelines.xhtml

telephone calls. Data on medication prescriptions, further investigations, hospitalisations and deaths will be extracted from participants' medical records.

Randomisation and blinding

Randomisation will be stratified by region with minimisation for age (6 to 23 months inclusive versus 2 to 12 years inclusive) and seasonal influenza vaccination status (yes or no/don't know). Randomisation is performed using Sortition®, an online clinical trial randomisation software package developed by the Clinical Trials Unit at the University of Oxford Nuffield Department of Primary Care Health Sciences. Health care professionals, the study team, children, and parents/guardians will all be blinded to treatment allocation.

Sample size

A large population-based study using the UK General Practice Research Database found that true complications occurred in 17.6% of at risk children aged 1 to 14 years within 30 days of being clinically diagnosed with influenza/ILI.(23) Assuming that true complications account for 44% of re-consultations due to clinical deterioration,(24) we estimate that 40% (17.6%/44 x 100) of at risk children with clinical influenza will re-consult with clinical deterioration within 30 days of initial presentation.

We will aim to recruit 650 children into the trial, including an inflation factor of 1.041 and allowance for 25% loss to follow-up. Our target effective sample size is therefore 484 children (242 in each arm), which would be sufficient to detect a reduction in re-consultation due to clinical deterioration from 40% to 26% (a 35% relative risk reduction) with 90% power and 5% two-tailed alpha error. Our inflation factor estimate is based on a conservative intracluster correlation estimate of 0.03(25) and a coefficient of variation of 0.6 (based on the value observed in the DD trial),(26) assuming an average cluster size of two patients.(27)

We acknowledge that recruitment of the above sample size is likely to be challenging (see Discussion). However, a sample of 280 participants would still be sufficient to detect a reduction in re-consultation due to clinical deterioration from 40% to 23% with 80% power and 5% two-tailed alpha error, including an inflation factor of 1.041 and allowance for 5% loss to follow-up. This effect size (a 42.5% relative risk reduction) is still conservative compared to the effect observed in the aforementioned trial conducted by Maeda et al.,(15) which reported an 85% relative risk reduction in incidence of pneumonia in children with ILI who were treated with the antibiotic sultamicillin (1/42 children) versus placebo (7/43 children). A 5% loss to follow-up rate should also be achievable given that data on re-consultations due to clinical deterioration will be extracted from medical notes.

Statistical analysis and economic evaluation plan

We will perform an intention-to-treat analysis including all randomised participants, as far as is practically possible, given any missing data. Specifically, the participants will be analysed in the groups to which they were allocated. Baseline characteristics will be summarised by treatment groups. The results from the trial will be presented as comparative summary statistics (difference in proportion or means) with 95% confidence intervals (CI). The analysis and reporting of results will follow the general principles of CONSORT 2010 statement.(28)

For peer review only - http://bmjopen.bmj.com/site/about/guidelines.xhtml

The proportion of children re-consulting due to clinical deterioration in the two groups (primary outcome) will be compared using a log-binomial regression model with adjustment for region, age and seasonal influenza vaccination status. The treatment effect will be reported as a relative risk with 95% confidence interval. A p-value will also be presented. Stability and assumptions of the regression model will be explored and alternative method will be used if any violation of assumptions occurred.

Analysis of continuous outcomes (e.g. duration of fever, duration of symptoms) will be compared regression analysis, adjusting for the same factors as described above. A nonparametric method will be used if assumptions of linear regression are not met. Binary outcomes (e.g. proportions of children prescribed medication and/or requiring further investigations, children in whom adverse events are reported, and children who are hospitalised or die within 28 days of randomisation) will be compared using Chi-squared/Fisher's exact test and log-binomial regression. Sensitivity analyses will be carried out to examine the robustness of the results with different assumptions about departures from randomisation policies, and handling of missing data.

The incremental costs of administering early treatment with co-amoxiclav in 'at risk' children with influenza/ILI versus placebo will be estimated using the data on the health care resource use provided by the four one-week study diaries and the medical notes review. We will estimate total costs and costs relating to burden on primary care, secondary care and parental/informal care. We will extrapolate our analysis of resource use and costs to explore the potential cost impact of early co-amoxiclav use on a national scale. The primary outcome measure for the cost-effectiveness analysis will be the EuroQol health-related quality of life youth proxy version (EQ-5D-Y proxy). We will estimate and report all the costs and consequences in a disaggregated format (cost-consequences analysis) as well as analysing and reporting the incremental cost and effectiveness in terms of cost per Quality-Adjusted Life Year (QALY) of administering co-amoxiclav versus placebo alongside a sensitivity analysis.

Safety and adverse event reporting

We will record all adverse events notified by trial investigators, health care professionals or participants as occurring within 28 days of randomisation, whether or not they are related to study medication. However, we will not undertake formal adverse event reporting for events which are known to be common side-effects of co-amoxiclav (i.e. mucocutaneous candidosis, diarrhoea, nausea and vomiting)(29)) unless they result in a serious adverse event or are considered by the child's clinician to be clinically severe.

The independent Data and Safety Monitoring Committee for the trial will be responsible for reviewing safety events after each recruitment season. The main aims of this review are:

- To ensure the safety of each trial participant;
- To identify any trends, such as increases in unexpected adverse events, and take appropriate action;
- To seek additional advice or information from investigators where required;
- To evaluate the risk of the trial continuing and take appropriate action where necessary;

For peer review only - http://bmjopen.bmj.com/site/about/guidelines.xhtml

- To act or advise, through the Chairperson or other consultant, on incidents occurring between meetings that require rapid assessment.

Patient and public involvement

To inform the design of our trial materials and procedures, we held discussion groups with 21 parents of children with ‘at risk’ conditions and 15 young people in four different locations (Oxford, London, Liverpool and Birmingham). These consultations informed the design of our ‘Archie the penguin’ mascot, study information leaflets, and study diaries (four one-week diaries instead of one diary to be completed over four weeks), as well as selection of the EQ-5D-Y proxy questionnaire to measure health-related quality of life in trial participants. They also prompted us to raise awareness about the study before the start of each recruitment season, produce a series of short videos on our study website to explain the study to parents and guardians, and initiate set-up of the home visit recruitment model in CRNs with the resources to support this.

DISCUSSION

Implications of study findings

The findings of the ARCHIE trial will provide important evidence to inform clinically appropriate and cost-effective antibiotic prescribing and use in ‘at risk’ children presenting with ILI in community health care settings. The only randomised placebo-controlled trial conducted to date of early antibiotic use in children with ILI(15) excluded children with chronic medical conditions (even though these children are at greatest risk of clinical deterioration) and did not evaluate potential implications of antibiotic treatment on antimicrobial resistance. There are also no studies to date which have examined the cost-effectiveness, costs and effectiveness of early antibiotic use in ‘at risk’ children with influenza/ILI.

The lack of clear, evidence-based guidance in this area results in considerable uncertainty and wide variation in clinical decision-making about prescribing antibiotics to children with ILI who have known risk factors for influenza-related clinical deterioration.(31) This may in turn result in antibiotics being prescribed unnecessarily, leading to emergence of antibiotic-resistant infections,(32) which are associated with worse clinical prognosis, even in patients with common respiratory tract infections in the community.(33)

Reflection

The ARCHIE trial is now in its fourth and final recruitment season. However, recruitment has proven to be more challenging than originally anticipated due to a significantly delayed start in recruitment, a limited pool of potential recruits, and limitations in capacity to recruit at participating sites.

We had originally planned to start recruitment in October 2014 and continue recruitment over three winter seasons (2014/15, 2015/16 and 2016/17). However, we were only able to start recruitment on 11th February 2015 due to delays in obtaining our study medication. By this time, the peak period of influenza/ILI consultation activity in early January 2015 had already passed.(34)

For peer review only - http://bmjopen.bmj.com/site/about/guidelines.xhtml

Extension of the UK childhood universal seasonal influenza vaccination programme to all children aged 4 years during winter 2014/15 and subsequent annual extension of that programme to older children may also have had an adverse impact on recruitment. Indeed, primary care consultation rates for influenza/ILI in England peaked at relatively modest levels during winters 2015/16 (28.7 per 100,000)(35) and 2016/17 (20.3 per 100,000)(36) compared to previous influenza seasons. The 2016/17 influenza season was particularly mild, with influenza/ILI consultation rates in England remaining above the Moving Epidemic Method baseline threshold of 14.3 per 100,000 for only six weeks.(36)

The highly specific nature of our study eligibility criteria also means that recruitment is only possible from a limited pool of children with one or more known 'at risk' condition (see box 1). Additionally, the health care professional assessing the child must be satisfied that the child does not require immediate antibiotic treatment. This has so far been a major barrier to recruitment, with the most common reasons for exclusion at the screening stage being receipt of antibiotics within the last 72 hours or requirement for immediate antibiotics. This suggests that parents and/or health care professionals were identifying specific reasons to treat these children with antibiotics, or erring on the side of caution by initiating antibiotic treatment in cases when they were unsure about clinical prognosis in the absence of treatment.

Staff at both primary care and hospital ambulatory care recruiting sites have reported difficulties with finding time to recruit participants alongside other work commitments at a time of increased seasonal health seeking activity. Due to the nature of ILI it is often not possible to anticipate when these children will present. Parents and children are sometimes unwilling to wait until suitably trained recruiting staff become available. Four-hour waiting time targets in hospital emergency departments mean that capacity to recruit is particularly limited in those settings, especially if there is no alternative space in which children can be assessed.

Strategies for addressing challenges encountered

To increase our pool of potential recruits, and compensate for our delayed start in recruitment, we have increased our total number of recruiting sites as far as staff capacity and resources will allow. To support efficient training of recruiting staff, we have produced a series of training videos on eligibility assessment, baseline clinical assessment, nasal and throat swab techniques, randomisation, study questionnaires and diaries, follow-up assessments (including adverse event reporting), and medical notes reviews, which can be accessed via our study website. Study expansion has also been facilitated by utilisation of existing infrastructure within the NIHR Clinical Research Networks (CRNs) to set up a home visit model for recruitment from general practices.

Recruitment via the home visit model started in winter 2016/17. This model only requires general practice staff to identify potentially eligible children before referring them to the study team, who then arrange for a research nurse to obtain informed consent and recruit children in their homes within 24 hours of initial presentation. This is therefore much less burdensome to individual general practices than recruiting participants on site.

We have also used CRN infrastructure to identify additional hospitals as recruiting sites, particularly focusing on hospitals with children's assessment units and direct access pathways which allow

For peer review only - http://bmjopen.bmj.com/site/about/guidelines.xhtml

families of children with known 'at risk' conditions to bring children directly to the children's assessment unit or ward instead of to the emergency department.

Additionally, we made three minor changes to our eligibility criteria in advance of starting recruitment in winter 2017/18. Our inclusion criteria now state that children who are permanently registered at a general practice in the UK (rather than in England only) are potentially eligible to participate. This has allowed us to set up the ARCHIE study in Wales, with the support of the Health and Care Research Wales Support Centre, which has experience of home visit recruitment for primary care clinical trials.(37) We have also clarified that children who have received antibiotics during the last 72 hours will only be excluded if these antibiotics were given to treat an acute infection. Children on long-term antibiotic prophylaxis are therefore still potentially suitable for inclusion in the study. Additionally, we have clarified that children who are felt to require immediate hospitalisation will only be excluded if this is specifically for treatment of an influenza-related complication or for a period of observation lasting longer than 24 hours.

Ethics and dissemination

The ARCHIE trial is registered on the International Standard Randomised Controlled Trials registry (ISRCTN 70714783) and European Clinical Trials Database (EudraCT: 2013-002822-21). We have obtained full ethical approval for this study from the North West - Liverpool East Research Ethics Committee (13/NW/0621), as well as approvals from the Health Research Authority and Medicines and Healthcare Products Regulatory Agency. All relevant amendments have been assessed and approved by REC/HRA and MHRA. Local R&D approvals/confirmation of capacity were received at all recruiting sites before recruitment commenced.

Full written informed consent will be obtained from a parent or guardian for each study participant. Children will also be invited to give written assent if appropriate. To ensure that their confidentiality is maintained, participants will be identified only by a participant identification number on all CRFs apart from the contact information sheet, consent form and assent form (if completed). All documents will be stored securely and will only be accessible by trial staff and authorised personnel. The study will comply with the Data Protection Act, which requires data to be anonymised as soon as it is practical to do so.

Our findings will be published in peer-reviewed journals and disseminated to patients and the public via our study website (www.archiestudy.com) and links with relevant charities representing children with 'at risk' conditions.

Authors' contributions: KW, TC and ST drafted the manuscript. KW is Chief Investigator of the ARCHIE trial. TC is the Senior Trial Manager and ST is the Trial Manager. MGS, ADH, MM, PL, CB, AF and AH contributed to development of the trial protocol and planning of recruitment strategies. MGS also provided input with aspects of the trial relating to microbiological assessments. MGS is the regional Principal Investigator (PI) of the trial in Liverpool, ADH is regional PI in Bristol, and MM is regional PI in Southampton. AH is the ARCHIE programme lead. LMY, SM and RP provided input with statistical aspects of the trial and developed the plan for statistical analysis of trial data. JW

For peer review only - http://bmjopen.bmj.com/site/about/guidelines.xhtml

developed the economic evaluation plan and is leading the economic evaluation of trial findings. All authors have reviewed and contributed edits to the manuscript.

Funding statement:

The ARCHIE trial is funded by the National Institute for Health Research (NIHR) under its Programme Grants for Applied Research Programme (Grant Reference RP-PG-1210-12012). The views expressed are those of the authors and not necessarily those of the UK National Health Service, the NIHR, or the UK Department of Health.

Competing interests statement:
Kay Wang is a NIHR Postdoctoral Fellow. Andrew Farmer is a NIHR Senior Investigator and is supported by the Oxford NIHR Biomedical Research Centre. Anthony Harnden is Deputy Chairman of the Joint Committee on Vaccination and Immunisation, which advises the UK government on vaccine policy.

References
1. Department of Health - Immunisation against infectious disease. Chapter 19: Influenza, 28 August 2015 Accessed at: https://www.gov.uk/government/uploads/system/uploads/attachment_data/file/456568/2904394_Green_Book_Chapter_19_v10_0.pdf (21st April 2017).
2. Prevention and Control of Seasonal Influenza with Vaccines. Recommendations of the Advisory Committee on Immunization Practices — United States, 2016–17 Influenza Season. Accessed 14th August 2017 at: https://www.cdc.gov/mmwr/volumes/65/rr/pdfs/rr6505.pdf.
3. Gill PJ, Ashdown HF, Wang K, Heneghan C, Roberts NW, Harnden A, et al. Identification of children at risk of influenza-related complications in primary and ambulatory care: a systematic review and meta-analysis. Lancet Respir Med. 2015;3(2):139-49.
4. Pitman RJ, Melegaro A, Gelb D, Siddiqui MR, Gay NJ, Edmunds WJ. Assessing the burden of influenza and other respiratory infections in England and Wales. J Infect. 2007;54:530-8.
5. Department of Health. Reference costs 2012-13. https://www.gov.uk/government/uploads/system/uploads/attachment_data/file/261154/nhs_reference_costs_2012-13_acc.pdf (accessed 2nd June 2014).
6. Curtis L. Unit Costs of Health and Social Care 2009 PSSRU, University of Kent at Canterbury; 2010.
7. Joint Committee on Vaccination and Immunisation - Minutes of the meeting held on Friday 13 April 2012. Accessed on 14th August 2017 at: http://media.dh.gov.uk/network/261/files/2012/05/JCVI-minutes-13-April-2012-meeting.pdf.
8. Public Health England. The National Childhood Flu Immunisation Programme 2017/18. Published July 2017. https://www.gov.uk/government/uploads/system/uploads/attachment_data/file/635920/Childhood_flu_programme_information_for_healthcare_practitioners.pdf (Accessed 20th November 2017).
9. Public Health England. Seasonal influenza vaccine uptake in children of primary school age: winter season 2016 to 2017. Final data for 1 September 2016 to 31 January 2017. Accessed on 17th August 2017 at: https://www.gov.uk/government/uploads/system/uploads/attachment_data/file/613457/Seasonal_influenza_vaccine_uptake_in_children_of_primary_school_age_2016_to_2017.pdf.
10. Rajaram S, Steffey A, Blak B, Hickman M, Christensen H, Caspard H. Uptake of childhood influenza vaccine from 2012-2013 to 2014-2015 in the UK and the implications for high-risk children: a retrospective observational cohort study. BMJ Open. 2016;6(8):e010625.

For peer review only - http://bmjopen.bmj.com/site/about/guidelines.xhtml

11. Pebody R, Warburton F, Ellis J, Andrews N, Potts A, Cottrell S, et al. Effectiveness of seasonal influenza vaccine for adults and children in preventing laboratory-confirmed influenza in primary care in the United Kingdom: 2015/16 end-of-season results. Euro Surveill. 2016;21(38):doi: 10.2807/1560-7917.es.2016.21.38.30348.
12. Jefferson T, Jones M, Doshi P, Spencer EA, Onakpoya I, Heneghan CJ. Oseltamivir for influenza in adults and children: systematic review of clinical study reports and summary of regulatory comments. BMJ. 2014;348:g2545.
13. Johnston SL, Ferrero F, Garcia ML, Dutkowski R. Oral oseltamivir improves pulmonary function and reduces exacerbation frequency for influenza-infected children with asthma. Pediatr Infect Dis J. 2005;24:225-32.
14. NICE. National Institute for Health and Clinical Excellence (NICE). Respiratory tract infections - antibiotic prescribing. NICE Clinical Guideline 69. London: Centre for Clinical Practice at NICE, 2008.
15. Maeda S, Yamada Y, Nakamura H, Maeda T. Efficacy of antibiotics against influenza-like illness in an influenza epidemic. Paediatr Int. 1999;41:274-6.
16. Harnden A, Perera R, Brueggemann AB, Mayon-White R, Crook DW, Thomson A, et al. Respiratory infections for which general practitioners consider prescribing an antibiotic: a prospective study. Arch Dis Child. 2007;92:594-7.
17. Klein EY, Monteforte B, Gupta A, Jiang W, May L, Hsieh YH, et al. The frequency of influenza and bacterial coinfection: a systematic review and meta-analysis. Influenza Other Respir Viruses. 2016;10(5):394-403.
18. McDanel JS, Perencevich EN, Storm J, Diekema DJ, Herwaldt L, Johnson JK, et al. Increased Mortality Rates Associated with Staphylococcus aureus and Influenza Co-infection, Maryland and Iowa, USA(1). Emerg Infect Dis. 2016;22(7):1253-6.
19. Wong KK, Jain S, Blanton L, Dhara R, Brammer L, Fry AM, et al. Influenza-associated pediatric deaths in the United States, 2004-2012. Pediatrics. 2013;132(5):796-804.
20. Goossens H, Ferech M, Vander Stichele R, Elseviers M. Outpatient antibiotic use in Europe and association with resistance: a cross-national database study. Lancet. 2005;365(9459):579-87.
21. National Institute for Health and Care Excellence (NICE). Antimicrobial stewardship: changing risk-related behaviours in the general population. NICE guideline [NG63] Published date: January 2017. https://www.nice.org.uk/guidance/ng63 (Accessed 20th November 2017).
22. Little P, Stuart B, Moore M, Coenen S, Butler CC, Godycki-Cwirko M, et al. Amoxicillin for acute lower respiratory tract infection in primary care when pneumonia is not suspected: a 12-country, randomised, placebo-controlled trial. Lancet Infect Dis. 2013;2013(13):123-9.
23. Meier CR, Napalkov PN, Wegmuller Y, Jefferson T, Jick H. Population-based study on incidence, risk factors, clinical complications and drug utilisation associated with influenza in the United Kingdom. Eur J Clin Microbiol Infect Dis. 2000;19:834-42.
24. Stott NCH. Management and outcome of winter upper respiratory tract infections in children aged 0-9 years. BMJ. 1979;1:29-31.
25. Adams G, Gulliford MC, Ukoumunne OC, Eldridge S, Chinn S, Campbell MJ. Patterns of intra-cluster correlation from primary care research to inform study design and analysis. J Clin Epidemiol. 2004;57:785-94.
26. Woodcock AJ, Kinmonth AL, Campbell MJ, Griffin SJ, Spiegal NM. Diabetes care from diagnosis: effects of training in patient-centred care on beliefs, attitudes and behaviour of primary care professionals. Patient Educ Couns. 1999;37:65-79.
27. Eldridge SM, Ashby D, Kerry S. Sample size for cluster randomized trials: the effect of coefficient of variation of cluster size and analysis method. Int J Epidemiol. 2006;35:1292-300.
28. Schulz KF, Altman DG, Moher D. CONSORT 2010 statement: updated guidelines for reporting parallel group randomised trials. BMJ. 2010;340:c332.
29. GlaxoSmithKline. Augmentin-Duo 400/57 - Summary of Product Characteristics. Accessed at http://www.medicines.org.uk/emcmobile/medicine/2026/spc on 25/03/2013. 2012.
30. MHRA. UKPAR Co-amoxiclav 457mg/5ml suspension (PL 21880/0011). 2009.

For peer review only - http://bmjopen.bmj.com/site/about/guidelines.xhtml

31. Ashdown HF, Raisanen U, Wang K, Ziebland S, Harnden A. Prescribing antibiotics to 'at-risk' children with influenza-like illness in primary care: qualitative study. BMJ Open. 2016;6(6):e011497.
32. Chung A, Perera R, Brueggemann AB, Elamin AE, Harnden A, Mayon-White R, et al. Effect of antibiotic prescribing on antibiotic resistance in individual children in primary care: prospective cohort study. BMJ. 2007;335:429.
33. van Hecke O, Wang K, Lee JJ, Roberts NW, Butler CC. Implications of Antibiotic Resistance for Patients' Recovery From Common Infections in the Community: A Systematic Review and Meta-analysis. Clin Infect Dis. 2017;65(3):371-82.
34. Public Health England. Surveillance of influenza and other respiratory viruses in the United Kingdom: winter 2014 to 2015. Accessed on 17th August 2017 at: https://www.gov.uk/government/uploads/system/uploads/attachment_data/file/429617/Annualreport_March2015_ver4.pdf.
35. Public Health England. Surveillance of influenza and other respiratory viruses in the United Kingdom: Winter 2015 to 2016. Accessed on 17th August 2017 at: https://www.gov.uk/government/uploads/system/uploads/attachment_data/file/526405/Flu_Annual_Report_2015_2016.pdf.
36. Public Health England. Surveillance of influenza and other respiratory viruses in the UK: Winter 2016 to 2017. Accessed on 17th August 2017 at: https://www.gov.uk/government/uploads/system/uploads/attachment_data/file/613493/Surveillance_of_influenza_and_other_respiratory_viruses_in_the_UK_2016_to_2017.pdf.
37. Francis NA, Ridd MJ, Thomas-Jones E, Butler CC, Hood K, Shepherd V, et al. Oral and Topical Antibiotics for Clinically Infected Eczema in Children: A Pragmatic Randomized Controlled Trial in Ambulatory Care. Ann Fam Med. 2017;15(2):124-30.

For peer review only

For peer review only - http://bmjopen.bmj.com/site/about/guidelines.xhtml

Appendix A: Laboratory analysis of throat swabs

Where possible, throat swabs will be obtained from participants at baseline for culture and sensitivity, and in a sub group at 3, 6 and 12 months. The swab will have a broth medium to improve retrieval of the target organisms, which are *Staphylococcus aureus*, *Streptococcus pneumoniae* and *Haemophilus influenzae*. Aliquots of the broth will be pipetted onto selective agar for each of the different organisms. A fourth plate, a selective agar for *Haemophilus influenzae* with ampicillin in the medium at 2mg/L will also be inoculated. Identification of target organisms will be performed in line with Public Health England Standards for Microbiological Investigation methods and susceptibility testing performed in accordance with the latest British Society of Antimicrobial Chemotherapy (BSAC) guidelines. The table below summarises the antimicrobials that will be used for susceptibility testing. Residual broth and isolates of the target organisms will then be put into long-term storage for potential further molecular analysis of resistance and determination of phenotypic resistance. The broth will be frozen to either -70°C or -80°C, in effect snap frozen. Thus, there will be no preservation of intact cells. Assessment of minimum inhibitory concentrations (MICs) will be performed using the agar stipulated in BSAC guidelines.

**Antimicrobial**	**Method**	***S.aureus***	***S.pneumoniae***	***H.influenzae***
Penicillin	MIC	No	Yes	No
Amoxicillin	MIC	No	No	Yes
Co-amoxiclav	MIC	Yes	Yes	Yes
Cefoxitin	MIC	Yes	No	No
Cefotaxime	MIC	No	Yes	Yes
Moxifloxacin	MIC	Yes	Yes	Yes
Erythromycin	MIC	Yes	Yes	No
Nalidixic acid	Disc 30µg	No	No	Yes

Table footnote:

MIC = Minimum Inhibitory Concentration
S. aureus = Staphylococcus aureus
S. pneumoniae = Streptococcus pneumoniae
H. influenzae = Haemophilus influenzae

For peer review only

Appendix B: Data collection

**Procedures**	**Enrolment**		**Post allocation (trial)**			**Close out**
	**V1**	**V2**	**Diary**	**T1**	**T2**	**NR**
Eligibility assessment	x					
Informed consent +/- assent		x				
Baseline assessment		x				
Nasal swab		x				
Throat swab*		x				
Randomisation		x				
Dispensing of study drug		x				
Allocation of study diary and pack		x				
Adverse events assessment				x	x	
Medical notes review						x
**Assessments**						
Age		x				
Sex		x				
Co-morbidity		x				x
Household smoking status		x				
Vaccination status		x				x
Antivirals/other medications		x				x
Regular medications						x
Heart rate		x				
Respiratory rate		x				
Baseline annual consultation rate						x
Re-consultations due to clinical deterioration						x
Duration of fever		x	x	x	x	
Duration of symptoms		x	x	x	x	
Further medications and/or further investigations						x
Adverse events				x	x	x
Hospitalisations/death				x	x	x
Health care resource use			x			x
EQ-5D-Y proxy		x	x			

V1 = Visit 1; V2 = Visit 2 (conducted within 24 hours of visit 1); T1 = telephone follow-up 1 (one week after randomisation); T2 = telephone follow-up 2 (two weeks after randomisation); NR = Notes Review (i.e. review of participant's medical record).

For peer review only

BMJ Open

The early use of Antibiotics for at Risk CHildren with InfluEnza (ARCHIE): protocol for a double-blind randomised placebo-controlled trial

Journal:	*BMJ Open*
Manuscript ID	bmjopen-2017-021144.R1
Article Type:	Protocol
Date Submitted by the Author:	15-Mar-2018
Complete List of Authors:	Wang, Kay; Nuffield Department of Primary Care Health Sciences<br>Carver, Tricia; University of Oxford, Nuffield Department of Primary Care Health Sciences<br>Tonner, Sharon; University of Oxford, Nuffield Department of Primary Care Health Sciences<br>Semple, Malcolm G; University of Liverpool, Women's and Children's Health, Institute of Translational Medicine<br>Hay, Alastair; University of Bristol, Centre for Academic Primary Care<br>Moore, Michael; University of Southampton Medical School, Primary Care Medical Group<br>Little, Paul; University of Southampton, Primary Care and Population Science<br>Butler, C; University of Oxford, Nuffield Department of Primary Care Health Sciences<br>Farmer, Andrew; University of Oxford, Nuffield Department of Primary Care Health Sciences<br>Perera, Rafael; University of Oxford, Nuffield Department of Primary Care Health Sciences<br>Yu, Ly-Mee; University of Oxford, Nuffield Department of Primary Care Health Sciences<br>Mallett, Susan; University of Birmingham, School of Health & Population Sciences<br>Wolstenholme, Jane; University of Oxford, Nuffield Department of Population Health<br>Harnden, Anthony; Oxford University, Nuffield Department of Primary Care Health Sciences
**Primary Subject Heading**:	Infectious diseases
Secondary Subject Heading:	General practice / Family practice, Paediatrics
Keywords:	Paediatric infectious disease & immunisation < PAEDIATRICS, Respiratory infections < THORACIC MEDICINE, Clinical trials < THERAPEUTICS

SCHOLARONE™
Manuscripts

For peer review only

The early use of Antibiotics for at Risk CHildren with InfluEnza (ARCHIE): protocol for a double-blind randomised placebo-controlled trial

Kay Wang[1], Tricia Carver[1], Sharon Tonner[1], Malcolm G Semple[2], Alastair D Hay[3], Michael Moore[4], Paul Little[4], Christopher Butler[1], Andrew Farmer[1], Rafael Perera[1], Ly-Mee Yu[1], Susan Mallett[5], Jane Wolstenholme[6], Anthony Harnden[1]

[1]Nuffield Department of Primary Care Health Sciences, University of Oxford, Radcliffe Primary Care, Woodstock Road, Oxford OX2 6GG, UK; [2]Women's and Children's Health, Institute of Translational Medicine, University of Liverpool, Alder Hey Children's Hospital, Eaton Road, Liverpool L12 2AP, UK; [3]University of Bristol, Centre for Academic Primary Care, Bristol Medical School: Population Health Sciences, Canynge Hall, 39 Whatley Road, Bristol BS8 2PS, UK; [4]Academic Unit: Primary Care and Population Sciences, University of Southampton, Aldermoor Health Centre, Aldermoor Close, Southampton SO16 5ST, UK; [5]Institute of Applied Health Research, University of Birmingham, Edgbaston, Birmingham B15 2TT, UK; [6]Health Economics Research Centre, Nuffield Department of Population Health, University of Oxford, Richard Doll Building, Old Road Campus, Oxford OX3 7LF, UK.

Kay Wang, NIHR Postdoctoral Fellow; Tricia Carver, Senior Trial Manager; Sharon Tonner, Trial Manager; Malcolm G Semple, Senior Lecturer in Child Health; Alastair Hay, Professor of Primary Care; Michael Moore, Professor in Primary Health Care Research; Paul Little, Professor of Primary Care Research; Christopher Butler, Professor of Primary Care; Andrew Farmer, Professor of General Practice; Rafael Perera, Professor of Medical Statistics; Ly-Mee Yu, Associate Professor; Susan Mallett, Senior Lecturer in Medical Statistics; Jane Wolstenholme, Associate Professor of Health Economics; Anthony Harnden, Professor of Primary Care.

Correspondence to: Kay Wang, kay.wang@phc.ox.ac.uk

For peer review only

For peer review only - http://bmjopen.bmj.com/site/about/guidelines.xhtml

ABSTRACT

Introduction. Influenza and influenza-like illness (ILI) create considerable burden on health care resources each winter. Children with pre-existing conditions such as asthma, diabetes mellitus, and cerebral palsy are amongst those at greatest risk of clinical deterioration from influenza/ILI. The ARCHIE trial aims to determine whether early oral treatment with the antibiotic co-amoxiclav reduces the likelihood of re-consultation due to clinical deterioration in these 'at risk' children.

Methods and Analysis: The ARCHIE trial is a double-blind parallel randomised placebo-controlled trial. 'At risk' children aged 6 months to 12 years inclusive who present within the first five days of an ILI episode will be randomised to receive a five-day course of oral co-amoxiclav 400/57 twice daily or placebo. Randomisation will use a non-deterministic minimisation algorithm to balance age and seasonal influenza vaccination status.

To detect respiratory virus infections, a nasal swab will be obtained from each participant before commencing study medication. To identify carriage of potential bacterial respiratory pathogens, we will also obtain a throat swab where possible.

The primary outcome is re-consultation in any health care setting due to clinical deterioration within 28 days of randomisation. We will analyse this outcome using log-binomial regression model adjusted for region, age and seasonal influenza vaccination status.

Secondary outcomes include duration of fever, duration of symptoms, and adverse events. Continuous outcomes will be compared using regression analysis (or equivalent nonparametric method for non-normal data) adjusting for minimisation variables. Binary outcomes will be compared using Chi-squared/Fisher's exact test and log-binomial regression.

Ethics: The ARCHIE trial has been reviewed and approved by the North West-Liverpool East Research Ethics Committee, Health Research Authority, and Medicines and Healthcare Products Regulatory Agency. Our findings will be published in peer-reviewed journals and disseminated via our study website (www.archiestudy.com) and links with relevant charities.

Trial Registration: ISRCTN 70714783; EudraCT: 2013-002822-21

For peer review only

For peer review only - http://bmjopen.bmj.com/site/about/guidelines.xhtml

STRENGTHS AND LIMITATIONS OF THIS STUDY

- This randomised controlled trial specifically focuses on 'at risk' children with known risk factors for complications from influenza/influenza-like illness (ILI), who may potentially benefit most from early antibiotic treatment.
- The primary outcome (re-consultation due to clinical deterioration within 28 days) is clinically relevant and has important implications for cost-effectiveness of early antibiotic treatment.
- The study uses a pragmatic case definition of ILI, which reflects current clinical practice, but does not reliably distinguish between influenza infection and ILI due to other causes.

For peer review only

For peer review only - http://bmjopen.bmj.com/site/about/guidelines.xhtml

INTRODUCTION

Background and rationale

Influenza is a viral infection which circulates mainly during winter and is a well-recognised risk factor for bacterial complications. ‘At risk’ children are more prone to becoming seriously unwell from influenza-related complications than otherwise healthy children.

‘At risk’ children are defined as children with underlying medical conditions or risk factors associated with an increased likelihood of developing complications from influenza/influenza-like illness (ILI). Based on guidance from the UK Department of Health(1) and the US Advisory Committee on Immunization Practices (ACIP),(2) ‘at risk’ groups include patients with chronic respiratory, cardiac, renal, liver and neurological conditions, as well as diabetes mellitus and immunosuppression. There is also systematic review evidence, mainly based on data from children under 2 years of age, that premature birth (i.e. delivery before 37 weeks gestation) is a risk factor for influenza-related complications.(3)

Based on data from Hospital Episode Statistics and the Office for National Statistics, 490,000 GP consultations and 4200 hospitalisations due to seasonal influenza occur each year in children aged 14 years or younger.(4) This results in a cost to the NHS of approximately £6.7 million due to hospitalisations(5) and £18 million due to primary care consultations.(6) The overall NHS and wider socioeconomic burden is likely to be greater due to additional costs incurred by critical care admissions, Accident and Emergency Department attendances, clinical interventions (investigations and medications) and parental productivity losses (days off work and childcare costs).

Based on recommendations from the Joint Committee on Vaccination and Immunisation,(7) the UK introduced universal seasonal influenza vaccination for all children aged 2 and 3 years from 2013/14, extending to all children aged 4 years during the 2014/15 influenza season. In 2017/18, Public Health England also initiated vaccination of children aged four to five years at school rather than in primary care, and included children in year 4 of school (aged eight to nine years) in the childhood influenza vaccination programme.(8)

Vaccine uptake of 55.7% has been observed in areas using school delivery models, compared to only 34.7% when general practice delivery models are used.(9) However, vaccination rates in children with ‘at risk’ conditions have not improved from around 40% since 2013/14.(10) Furthermore, effectiveness of the UK 2015/16 seasonal influenza vaccination was only around 58% in children aged 2 to 17 years.(11) Evidence for treatment with neuraminidase inhibitors in ‘at risk’ children is also weak. Oseltamivir has not been demonstrated to provide significant benefit in terms of shortening symptom duration(12) or reducing acute exacerbations(13) in children with asthma.

Although there is a substantial evidence base underpinning recommendations that routine antibiotic treatment is not indicated for viral respiratory tract infections,(14) there is also preliminary evidence to suggest that early antibiotic use may be beneficial in preventing clinical deterioration and complications due to influenza. The results of a small randomised placebo-controlled trial suggest that early treatment with the antibiotic sultamicillin in children presenting with influenza/ILI significantly reduces the incidence of pneumonia.(15) Additionally, observational data from school aged children presenting in primary care with cough and fever found that duration of fever was

For peer review only - http://bmjopen.bmj.com/site/about/guidelines.xhtml

significantly shorter in children with laboratory-confirmed influenza who received antibiotics (mostly amoxicillin) at an early stage during their illness.(16)

In recognition of the potentially serious clinical and socioeconomic consequences of bacterial complications of influenza, the UK government stockpiles the antibiotic co-amoxiclav for use during influenza epidemics and pandemics. Co-amoxiclav is the antibiotic of choice due to its coverage of both *Streptococcus pneumoniae* and *Staphylococcus aureus*, the most commonly observed bacterial co-infections in patients with laboratory-confirmed influenza.(17) In particular, *Staphylococcus aureus* co-infections are associated with worse clinical outcomes in patients with laboratory-confirmed influenza(18) and were found in nearly half of influenza-related deaths between October 2004 and September 2012 among US children from whom at least one bacterial pathogen was identified from a normally sterile site.(19)

At the same time, antibiotic prescribing is a major driver of antimicrobial resistance,(20) which is well recognised as an emerging threat to the treatment of serious bacterial infections. Data on the potential impact of early co-amoxiclav use on development and duration of antimicrobial resistance are therefore needed to guide future national guidance on supply and use of this antibiotic during periods of high influenza activity. A clear, evidence-based understanding of the benefits versus harms will in turn support local antibiotic stewardship programmes and better informed, more appropriate decisions about antibiotic prescribing and use in the community.(21)

METHODS AND ANALYSIS

Objectives

Our primary objective is to determine whether early treatment with co-amoxiclav reduces the likelihood of re-consultation due to clinical deterioration in 'at risk' children with influenza/influenza-like illness (ILI).

Other objectives are:

- To determine whether early treatment with co-amoxiclav reduces duration of fever in 'at risk' children with influenza/ILI.
- To determine whether early treatment with co-amoxiclav reduces duration of symptoms in 'at risk' children with influenza/ILI.
- To compare further intervention rates in 'at risk' children with influenza/ILI treated with co-amoxiclav versus placebo.
- To compare adverse events in 'at risk' children with influenza/ILI treated with co-amoxiclav versus placebo.
- To assess the cost, outcomes and cost-effectiveness of early treatment with co-amoxiclav in 'at risk' children with influenza/ILI versus placebo.
- To determine the impact on long-term respiratory bacterial carriage and antibiotic resistance of early treatment with co-amoxiclav versus placebo in 'at risk' children with influenza/ILI.

Study design

For peer review only - http://bmjopen.bmj.com/site/about/guidelines.xhtml

The ARCHIE study is a multi-centre double-blind randomised placebo-controlled trial. 'At risk' children (i.e. children with known risk factors for influenza-related complications) who present within the first five days of an influenza-like illness and who are not considered by their clinician to require immediate antibiotic treatment or hospitalisation, will be randomised to receive a 5-day course of co-amoxiclav 400/57 mg or a matching placebo. Nasal swabs will be obtained from all participants to detect influenza infection. Throat swabs for culture and sensitivity will also be obtained at baseline where possible. The primary outcome is re-consultation due to clinical deterioration within 28 days of randomisation.

Study participants

We will recruit 'at risk' children aged six months to 12 years inclusive who present within the first five days of an influenza-like illness (ILI). We will define ILI as the presence of both cough and fever, which may be defined as child-reported fever, parent-reported fever or temperature >37.8°C (axillary or tympanic temperature measurement). This is intended as a pragmatic case definition, which can be reliably applied across our entire target age range.

Box 1 summarises our definition of 'at risk' groups. Rather than being an exhaustive list, this definition is intended to guide clinicians in identifying which children are likely to be at greater risk of influenza-related clinical deterioration or complications. Health care professionals are also advised to use their clinical judgement to identify 'at risk' children. Children with other potential risk factors who may be suitable to take part may be discussed with a medically qualified member of the research team. Children who require immediate antibiotics or hospital admission for treatment of an influenza-related complication (based on their clinician's judgement) will not be eligible to take part. Box 2 summarises our full inclusion and exclusion criteria.

BOX 1: 'AT RISK' GROUPS

Respiratory

- Asthma requiring continuous or repeated use of controller therapy (e.g. inhaled steroids, leukotriene receptor antagonists, long-acting beta agonists, systemic steroids).
- Admitted to hospital with exacerbation of asthma within the last 12 months.
- Admitted to hospital with bronchiolitis or pneumonia within the last 12 months.
- Recurrent viral wheeze (3 or more episodes within the last 12 months).
- Bronchopulmonary dysplasia.

Cardiac

- Congenital heart disease being actively managed or monitored by cardiology team.
- Chronic heart failure being actively managed or monitored by cardiology team.

Neurological

- Chronic neurological or neuromuscular disorder which compromises respiratory function (e.g. cerebral palsy).

Renal

- Chronic kidney disease defined as either of the following:
- Impaired eGFR[§] (estimated glomerular filtration rate) measurement within the last 12 months.

For peer review only - http://bmjopen.bmj.com/site/about/guidelines.xhtml

- Known hereditary or structural kidney abnormality with or without impairment in eGFR.
- Nephrotic syndrome.
- Kidney transplantation.

Liver[§§]

- Cirrhosis
- Biliary atresia
- Chronic hepatitis

Immunodeficiency

- Asplenia or splenic dysfunction.
- HIV infection.
- Undergoing chemotherapy leading to immunosuppression.
- Taking systemic steroids at a dose equivalent to prednisolone 20mg or more per day (any age) or >=1mg per kg per day (children under 20kg).

Other

- Diabetes mellitus (type 1 or type 2) or other metabolic condition.
- Genetic abnormality (e.g. Down's syndrome)
- Sickle cell disease
- Malignancy
- Prematurity (born before 37 weeks gestation) in children aged 6 to 23 months.

[§]Impaired eGFR is defined as an eGFR measurement of 59 ml/min/1.73m^2 or less within the last 12 months before study entry. However, to enter the trial the following two conditions must also be satisfied:

1) eGFR >=30 ml/min/1.73m^2 based on most recent measurement within the last 12 months;
2) no reason to suspect further deterioration in eGFR at time of study entry.

[§§]Children with mild or moderate liver disease may enter the trial. However, to minimise the risk of serious hepatic complications related to study medication, children with severe liver disease may not enter the trial. Severe liver disease is defined as hepatic impairment associated with any of the following: jaundice, impaired coagulation/increased bleeding risk, bilirubin persistently greater than 50 micromol/litre (two measurements within last 12 months).

BOX 2: Study inclusion and exclusion criteria

Inclusion Criteria

- Aged 6 months to 12 years inclusive.
- In 'at risk' category.
- Presenting with influenza-like illness (i.e. cough and fever) during influenza season.
- Presenting within 5 days of symptom onset.
- Permanently registered at a general practice in UK.
- Parent /guardian able to complete study diary and questionnaires.

For peer review only - http://bmjopen.bmj.com/site/about/guidelines.xhtml

Exclusion Criteria

The participant may not enter the trial if ANY of the following apply:

- Known contraindication to co-amoxiclav.
- Child given antibiotics for treatment of an acute infection within the last 72 hours.
- Child requires immediate antibiotics (clinician's judgement).
- Child requires immediate hospital admission for treatment of an influenza-related complication (clinician's judgement).
- Child has been observed on hospital ward or ambulatory care unit for longer than 24 hours.
- Presence of any reason to prevent healthcare professional from obtaining nasal swab.
- Child with known cystic fibrosis.
- Child previously entered into the ARCHIE study.
- Child has been involved in another medicinal trial within the last 90 days.

Recruitment

We will recruit study participants from a range of health care settings where 'at risk' children from the community present with influenza/ILI. Recruiting sites will include general practices, walk-in centres and hospitals. Identification of participants at these sites may be supported by the use of participant identification centres. Given the seasonal nature with which influenza circulates in the community, each recruitment season will commence at the beginning of October and continue until the end of March the following year, or later if influenza continues to circulate above seasonal thresholds.

Before each recruitment season, we will ask participating general practices to perform database searches to identify children within our target age range who are in 'at risk' groups. A medically qualified individual will manually screen the results of the database search to exclude any children who would not be suitable to invite to take part in the study. Recruiting sites may also install a study reminder screen prompt, which will appear when the electronic medical records of potentially suitable children are accessed. Participating hospitals will also be asked to identify potential recruits through specialist outpatient clinics which manage children with 'at risk' conditions.

To further raise awareness about the trial and opportunities for participation, we will provide recruiting sites with study promotional materials including posters and short information leaflets, which summarise our study aims and direct families of potential participants to our study website (www.archiestudy.com). We will also liaise with recruiting sites to raise awareness via local news outlets, social media, and charities representing relevant patient groups. Parents who express an interest in allowing their child to take part in the study will be able to contact the study team or look at the study website to find their nearest recruiting site.

During each recruitment season, children who present at recruiting sites with influenza/ILI will be screened to determine whether they are eligible to take part in the trial. Suitably trained health care professionals will then obtain informed consent for study participation from the child's parent

For peer review only - http://bmjopen.bmj.com/site/about/guidelines.xhtml

or guardian and complete study enrolment and randomisation procedures either at the recruiting site or at the child's home within 24 hours of confirming their eligibility for study participation.

Intervention

Participants will be randomised to receive either co-amoxiclav 400/57 (amoxicillin 400 mg as trihydrate and clavulanic acid 57 mg as potassium salt/5 mL when reconstituted with water) or a matching placebo, which will be taken orally twice daily for 5 days. Study medication doses based on age, as well as weight in participants aged 6 to 23 months, will be calculated according to the standard dosing regimen for co-amoxiclav 400/57 recommended by the British National Formulary (BNF, see box 3). Study medication will be provided as a dry powder, which health care professionals will reconstitute with water once the participant has been randomised.

Medically qualified individuals will use their clinical judgement when advising on study medication doses for any children to whom they feel that standard BNF dosing recommendations should not apply.

BOX 3: Study medication dosing regimen

**Child's age**	**Study medication dose**
**6 months to 23 months**	
Under 6 kg	Calculate dose according to British National Formulary instructions for co-amoxiclav 400/57. Advise two doses daily for 5 days.
6.0 – 7.9 kg	1 ml twice daily for 5 days
8.0 – 10.9 kg	1.5 ml twice daily for 5 days
11.0 – 12.9 kg	2 ml twice daily for 5 days
**2 to 6 years**	2.5 ml twice daily for 5 days
**7 to 12 years**	5 ml twice daily for 5 days

Participants will be allowed to continue their usual regular medications and any additional medications for their influenza/ILI episode while taking part in the trial.

Baseline study procedures

Health care professionals will record baseline data from study participants on age, sex, co-morbidities, household smoking status, influenza vaccination status, medications given during the current illness episode, duration of fever, and duration of symptoms. Heart rate and respiratory rate will also be measured and recorded at baseline. Other baseline characteristics (medical conditions, regular medications, vaccinations, and acute consultations which occurred up to 12 months before randomisation) will be extracted from participants' medical records.

For peer review only - http://bmjopen.bmj.com/site/about/guidelines.xhtml

For peer review only

A nasal swab will be obtained from each participant at baseline. Nasal swabs will be placed in viral transport medium and sent by post to Alder Hey Children's Hospital microbiology department for analysis, where they will be analysed by real-time Polymerase Chain Reaction (PCR) analysis to detect influenza and distinguish influenza A, B and A/H1N1 2009 pandemic subtypes. Residual medium will be retained for potential future detection of other pathogens. Where possible, a throat swab will also be obtained from the participant for culture and sensitivity. In a subgroup of participants whose parents/guardians give additional written informed consent, further throat swabs will be collected after three, six and twelve months. Appendix A provides further details of the laboratory analyses which will be performed on throat swab samples.

Outcomes

The primary outcome is re-consultation due to clinical deterioration within 28 days of randomisation. Clinical deterioration is defined as any of: worsening symptoms, development of new symptoms or development of a complication requiring medication or hospitalisation. This definition is based on that used by the GRACE (Genomics to combat Resistance against Antibiotics in Community-acquired lower respiratory tract infection in Europe) consortium in to define re-consultations due to clinical deterioration in patients with lower respiratory tract infections.(22)

Secondary outcomes include duration of fever and duration of symptoms from time of randomisation, and medication prescriptions and/or further investigations, adverse events, hospitalisations or deaths, all within 28 days of randomisation. For the purpose of the economic evaluation, outcomes include healthcare resource utilisation and parental/informal care costs within 28 days from randomisation and health related quality of life as measured by the EQ-5D-Y proxy at baseline and days 4, 7, 14 and 28.

Data collection

Appendix B presents a complete summary of data collected during the trial. Data obtained at the baseline assessment, week 1 and week 2 follow-ups, as well as data on safety events, will be entered on paper case report forms before being sent to the main trial office for entry using OpenClinica open source software, version 3.13 Waltham MA, USA, a validated online electronic data capture system. All data will be double entered to ensure accuracy. Data extracted from the medical notes of children recruited from general practices will be directly transcribed into OpenClinica at the child's general practice. The main trial office will contact the general practices of children recruited from hospitals or walk-in centres to request relevant data from their medical notes, which will then be entered by a member of the trial team.

A parent or guardian will be asked to complete and return by post a series of four one-week study diaries on behalf of the participant to provide data on doses of study medication given to the child, axillary temperature, symptoms, adverse events, daily activities and childcare, child's health-related quality of life measured by the EQ-5D-Y proxy and health service contacts such as hospitalisations and visits to the GP. Health care professionals will also contact each participant's parent or guardian by telephone one week and two weeks after randomisation to collect data on duration of fever, adverse events, and adherence to study medication. Primary outcome data will be extracted from participants' medical notes at least three months after randomisation. This is to allow records of any hospital admissions or consultations in non-general practice settings to be captured in the child's

For peer review only - http://bmjopen.bmj.com/site/about/guidelines.xhtml

general practice medical record. Data on duration of symptoms will be collected from study diaries completed by participants' parents/guardians. Data on duration of fever will also be collected from study diaries and supplemented by follow-up telephone calls with parents/guardians one week and two weeks after randomisation. During these telephone calls, parents/guardians will be asked whether or not their child's fever has settled and if so, when. Data on adverse events will also be collected at these telephone calls. Data on medication prescriptions, further investigations, hospitalisations and deaths will be extracted from participants' medical records.

Randomisation and blinding

Randomisation will be stratified by region with minimisation for age (6 to 23 months inclusive versus 2 to 12 years inclusive) and seasonal influenza vaccination status (yes or no/don't know). Randomisation is performed using Sortition®, an online clinical trial randomisation software package developed by the Clinical Trials Unit at the University of Oxford Nuffield Department of Primary Care Health Sciences. The randomisation system will be implemented and managed by the Clinical Trials Unit. The randomisation codes will be maintained by a specifically appointed independent custodian. Health care professionals, the study team, children, and parents/guardians will all be blinded to treatment allocation.

A participant's treatment allocation will be unblinded in the event of a suspected unexpected serious adverse reaction (SUSAR). Where there is a perceived need for unblinding, the participant's responsible clinician (i.e. the clinician treating the participant) will discuss the case with the Chief Investigator or a designated alternative study clinician. If it is agreed that unblinding is required, the Chief Investigator or designated study clinician will request that the randomisation code is accessed by the independent custodian, who will disclose the participant's treatment allocation to his/her responsible clinician.

Sample size

A large population-based study using the UK General Practice Research Database found that true complications occurred in 17.6% of at risk children aged 1 to 14 years within 30 days of being clinically diagnosed with influenza/ILI.(23) Assuming that true complications account for 44% of re-consultations due to clinical deterioration,(24) we estimate that 40% (17.6%/44 x 100) of at risk children with clinical influenza will re-consult with clinical deterioration within 30 days of initial presentation.

We will aim to recruit 650 children into the trial, including an inflation factor of 1.041 and allowance for 25% loss to follow-up. Our target effective sample size is therefore 484 children (242 in each arm), which would be sufficient to detect a reduction in re-consultation due to clinical deterioration from 40% to 26% (a 35% relative risk reduction) with 90% power and 5% two-tailed alpha error. Our inflation factor estimate is based on a conservative intracluster correlation estimate of 0.03(25) and a coefficient of variation of 0.6 (based on the value observed in the DD trial),(26) assuming an average cluster size of two patients.(27)

We acknowledge that recruitment of the above sample size is likely to be challenging (see Discussion). However, a sample of 280 participants would still be sufficient to detect a reduction in re-consultation due to clinical deterioration from 40% to 23% with 80% power and 5% two-tailed

For peer review only - http://bmjopen.bmj.com/site/about/guidelines.xhtml

alpha error, including an inflation factor of 1.041 and allowance for 5% loss to follow-up. This effect size (a 42.5% relative risk reduction) is still conservative compared to the effect observed in the aforementioned trial conducted by Maeda et al.,(15) which reported an 85% relative risk reduction in incidence of pneumonia in children with ILI who were treated with the antibiotic sultamicillin (1/42 children) versus placebo (7/43 children). A 5% loss to follow-up rate should also be achievable given that data on re-consultations due to clinical deterioration will be extracted from medical notes.

Statistical analysis and economic evaluation plan

We will perform an intention-to-treat analysis including all randomised participants and use multiple imputation methods to account for missing data. Specifically, the participants will be analysed in the groups to which they were allocated. Baseline characteristics will be summarised by treatment groups. The results from the trial will be presented as comparative summary statistics (difference in proportion or means) with 95% confidence intervals (CI). The analysis and reporting of results will follow the general principles of CONSORT 2010 statement.(28)

The proportion of children re-consulting due to clinical deterioration in the two groups (primary outcome) will be compared using a log-binomial regression model with adjustment for region, age and seasonal influenza vaccination status. The treatment effect will be reported as a relative risk with 95% confidence interval. A p-value will also be presented. Stability and assumptions of the regression model will be explored and alternative method will be used if any violation of assumptions occurred.

Analysis of continuous outcomes (e.g. duration of fever, duration of symptoms) will be compared regression analysis, adjusting for the same factors as described above. A nonparametric method will be used if assumptions of linear regression are not met. Binary outcomes (e.g. proportions of children prescribed medication and/or requiring further investigations, children in whom adverse events are reported, and children who are hospitalised or die within 28 days of randomisation) will be compared using Chi-squared/Fisher's exact test and log-binomial regression. Sensitivity analyses will be carried out to examine the robustness of the results with different assumptions about departures from randomisation policies, and handling of missing data.

The incremental costs of administering early treatment with co-amoxiclav in 'at risk' children with influenza/ILI versus placebo will be estimated using the data on the health care resource use provided by the four one-week study diaries and the medical notes review. We will estimate total costs and costs relating to burden on primary care, secondary care and parental/informal care. We will extrapolate our analysis of resource use and costs to explore the potential cost impact of early co-amoxiclav use on a national scale. The primary outcome measure for the cost-effectiveness analysis will be the EuroQol health-related quality of life youth proxy version (EQ-5D-Y proxy). We will estimate and report all the costs and consequences in a disaggregated format (cost-consequences analysis) as well as analysing and reporting the incremental cost and effectiveness in terms of cost per Quality-Adjusted Life Year (QALY) of administering co-amoxiclav versus placebo alongside a sensitivity analysis.

Safety and adverse event reporting

For peer review only - http://bmjopen.bmj.com/site/about/guidelines.xhtml

We will record all adverse events notified by trial investigators, health care professionals or participants as occurring within 28 days of randomisation, whether or not they are related to study medication. However, we will not undertake formal adverse event reporting for events which are known to be common side-effects of co-amoxiclav (i.e. mucocutaneous candidosis, diarrhoea, nausea and vomiting)(29)) unless they result in a serious adverse event or are considered by the child's clinician to be clinically severe.

Clinicians will be advised to discontinue a participant's study medication if he/she experiences an adverse drug reaction related to the study medication. Parents/guardians of participants whose study medication is discontinued will still be requested to complete their study diaries and questionnaires and will still receive telephone follow-up calls unless they choose to withdraw consent for these.

The independent Data and Safety Monitoring Committee (DSMC) for the trial will be responsible for reviewing safety events after each recruitment season. The main aims of this review are:

- To ensure the safety of each trial participant;
- To identify any trends, such as increases in unexpected adverse events, and take appropriate action;
- To seek additional advice or information from investigators where required;
- To evaluate the risk of the trial continuing and take appropriate action where necessary;
- To act or advise, through the Chairperson or other consultant, on incidents occurring between meetings that require rapid assessment.

The DSMC will also advise on whether the trial should be terminated based on its reviews of serious adverse events in consultation with the Trial Steering Committee and trial Sponsor if necessary.

Patient and public involvement

Our patient advisers and patient representatives identified via the NIHR Medicines for Children Research Network Clinical Study Groups helped develop and refine our study objectives by providing extensive feedback on early draft versions of our research plan. To inform the design of our trial materials and procedures, we held discussion groups with 21 parents of children with 'at risk' conditions and 15 young people in four different locations (Oxford, London, Liverpool and Birmingham). These consultations informed the design of our 'Archie the penguin' mascot, study information leaflets, and study diaries (four one-week diaries instead of one diary to be completed over four weeks), as well as selection of the EQ-5D-Y proxy questionnaire to measure health-related quality of life in trial participants. They also prompted us to raise awareness about the study before the start of each recruitment season, produce a series of short videos on our study website to explain the study to parents and guardians, and initiate set-up of the home visit recruitment model in CRNs with the resources to support this. Patient representatives will also help us disseminate our research findings by providing assistance with writing plain language summaries and establishing links with relevant charities and patient organisations which represent parents and children to whom the findings of our research will be relevant.

For peer review only - http://bmjopen.bmj.com/site/about/guidelines.xhtml

DISCUSSION

Implications of study findings

The findings of the ARCHIE trial will provide important evidence to inform clinically appropriate and cost-effective antibiotic prescribing and use in 'at risk' children presenting with ILI in community health care settings. The only randomised placebo-controlled trial conducted to date of early antibiotic use in children with ILI(15) excluded children with chronic medical conditions (even though these children are at greatest risk of clinical deterioration) and did not evaluate potential implications of antibiotic treatment on antimicrobial resistance. There are also no studies to date which have examined the cost-effectiveness, costs and effectiveness of early antibiotic use in 'at risk' children with influenza/ILI.

The lack of clear, evidence-based guidance in this area results in considerable uncertainty and wide variation in clinical decision-making about prescribing antibiotics to children with ILI who have known risk factors for influenza-related clinical deterioration.(30) This may in turn result in antibiotics being prescribed unnecessarily, leading to emergence of antibiotic-resistant infections,(31) which are associated with worse clinical prognosis, even in patients with common respiratory tract infections in the community.(32)

Reflection

The ARCHIE trial is now in its fourth and final recruitment season. However, recruitment has proven to be more challenging than originally anticipated due to a significantly delayed start in recruitment, a limited pool of potential recruits, and limitations in capacity to recruit at participating sites.

We had originally planned to start recruitment in October 2014 and continue recruitment over three winter seasons (2014/15, 2015/16 and 2016/17). However, we were only able to start recruitment on 11th February 2015 due to delays in obtaining our study medication. By this time, the peak period of influenza/ILI consultation activity in early January 2015 had already passed.(33)

Extension of the UK childhood universal seasonal influenza vaccination programme to all children aged 4 years during winter 2014/15 and subsequent annual extension of that programme to older children may also have had an adverse impact on recruitment. Indeed, primary care consultation rates for influenza/ILI in England peaked at relatively modest levels during winters 2015/16 (28.7 per 100,000)(34) and 2016/17 (20.3 per 100,000)(35) compared to previous influenza seasons. The 2016/17 influenza season was particularly mild, with influenza/ILI consultation rates in England remaining above the Moving Epidemic Method baseline threshold of 14.3 per 100,000 for only six weeks.(35) However, previous research suggests that GPs rarely consider influenza infection or ask about vaccination status when assessing 'at risk' children with ILI.(30) It is therefore unlikely that recruitment would have been significantly compromised due to health care professionals failing to consider vaccinated children as potential recruits.

The highly specific nature of our study eligibility criteria also means that recruitment is only possible from a limited pool of children with one or more known 'at risk' condition (see box 1). Additionally, the health care professional assessing the child must be satisfied that the child does not require immediate antibiotic treatment. This has so far been a major barrier to recruitment, with the most common reasons for exclusion at the screening stage being receipt of antibiotics within the last 72

For peer review only - http://bmjopen.bmj.com/site/about/guidelines.xhtml

hours or requirement for immediate antibiotics. This suggests that parents and/or health care professionals were identifying specific reasons to treat these children with antibiotics, or erring on the side of caution by initiating antibiotic treatment in cases when they were unsure about clinical prognosis in the absence of treatment.

Staff at both primary care and hospital ambulatory care recruiting sites have reported difficulties with finding time to recruit participants alongside other work commitments at a time of increased seasonal health seeking activity. Due to the nature of ILI it is often not possible to anticipate when these children will present. Parents and children are sometimes unwilling to wait until suitably trained recruiting staff become available. Four-hour waiting time targets in hospital emergency departments mean that capacity to recruit is particularly limited in those settings, especially if there is no alternative space in which children can be assessed.

Strategies for addressing challenges encountered

To increase our pool of potential recruits, and compensate for our delayed start in recruitment, we have increased our total number of recruiting sites as far as staff capacity and resources will allow. To support efficient training of recruiting staff, we have produced a series of training videos on eligibility assessment, baseline clinical assessment, nasal and throat swab techniques, randomisation, study questionnaires and diaries, follow-up assessments (including adverse event reporting), and medical notes reviews, which can be accessed via our study website. Study expansion has also been facilitated by utilisation of existing infrastructure within the NIHR Clinical Research Networks (CRNs) to set up a home visit model for recruitment from general practices.

Recruitment via the home visit model started in winter 2016/17. This model only requires general practice staff to identify potentially eligible children before referring them to the study team, who then arrange for a research nurse to obtain informed consent and recruit children in their homes within 24 hours of initial presentation. This is therefore much less burdensome to individual general practices than recruiting participants on site.

We have also used CRN infrastructure to identify additional hospitals as recruiting sites, particularly focusing on hospitals with children's assessment units and direct access pathways which allow families of children with known 'at risk' conditions to bring children directly to the children's assessment unit or ward instead of to the emergency department.

Additionally, we made three minor changes to our eligibility criteria in advance of starting recruitment in winter 2017/18. Our inclusion criteria now state that children who are permanently registered at a general practice in the UK (rather than in England only) are potentially eligible to participate. This has allowed us to set up the ARCHIE study in Wales, with the support of the Health and Care Research Wales Support Centre, which has experience of home visit recruitment for primary care clinical trials.(36) We have also clarified that children who have received antibiotics during the last 72 hours will only be excluded if these antibiotics were given to treat an acute infection. Children on long-term antibiotic prophylaxis are therefore still potentially suitable for inclusion in the study. Additionally, we have clarified that children who are felt to require immediate hospitalisation will only be excluded if this is specifically for treatment of an influenza-related complication or for a period of observation lasting longer than 24 hours.

For peer review only - http://bmjopen.bmj.com/site/about/guidelines.xhtml

Ethics and dissemination

The ARCHIE trial is registered on the International Standard Randomised Controlled Trials registry (ISRCTN 70714783) and European Clinical Trials Database (EudraCT: 2013-002822-21). We have obtained full ethical approval for this study from the North West - Liverpool East Research Ethics Committee (13/NW/0621), as well as approvals from the Health Research Authority and Medicines and Healthcare Products Regulatory Agency. All relevant amendments have been assessed and approved by REC/HRA and MHRA. Local R&D approvals/confirmation of capacity were received at all recruiting sites before recruitment commenced.

The trial will be conducted in accordance with the principles of Good Clinical Practice (GCP) and relevant regulations. Full written informed consent will be obtained from a parent or guardian for each study participant. Children will also be invited to give written assent if appropriate. To ensure that their confidentiality is maintained, participants will be identified only by a participant identification number on all CRFs apart from the contact information sheet, consent form and assent form (if completed). All documents will be stored securely and will only be accessible by trial staff and authorised personnel. The study will comply with the Data Protection Act, which requires data to be anonymised as soon as it is practical to do so.

Our findings will be published in peer-reviewed journals and disseminated to patients and the public via our study website (www.archiestudy.com) and links with relevant charities representing children with 'at risk' conditions.

References

1. Department of Health - Immunisation against infectious disease. Chapter 19: Influenza, 28 August 2015 Accessed at: https://www.gov.uk/government/uploads/system/uploads/attachment_data/file/456568/2904394_Green_Book_Chapter_19_v10_0.pdf (21st April 2017).

2. Prevention and Control of Seasonal Influenza with Vaccines. Recommendations of the Advisory Committee on Immunization Practices — United States, 2016–17 Influenza Season. Accessed 14th August 2017 at: https://www.cdc.gov/mmwr/volumes/65/rr/pdfs/rr6505.pdf.

3. Gill PJ, Ashdown HF, Wang K, Heneghan C, Roberts NW, Harnden A, et al. Identification of children at risk of influenza-related complications in primary and ambulatory care: a systematic review and meta-analysis. Lancet Respir Med. 2015;3(2):139-49.

4. Pitman RJ, Melegaro A, Gelb D, Siddiqui MR, Gay NJ, Edmunds WJ. Assessing the burden of influenza and other respiratory infections in England and Wales. J Infect. 2007;54:530-8.

5. Department of Health. Reference costs 2012-13. https://www.gov.uk/government/uploads/system/uploads/attachment_data/file/261154/nhs_reference_costs_2012-13_acc.pdf (accessed 2nd June 2014).

6. Curtis L. Unit Costs of Health and Social Care 2009 PSSRU, University of Kent at Canterbury; 2010.

7. Joint Committee on Vaccination and Immunisation - Minutes of the meeting held on Friday 13 April 2012. Accessed on 14th August 2017 at: http://media.dh.gov.uk/network/261/files/2012/05/JCVI-minutes-13-April-2012-meeting.pdf.

For peer review only - http://bmjopen.bmj.com/site/about/guidelines.xhtml

8. Public Health England. The National Childhood Flu Immunisation Programme 2017/18. Published July 2017. https://www.gov.uk/government/uploads/system/uploads/attachment_data/file/635920/Childhood_flu_programme_information_for_healthcare_practitioners.pdf (Accessed 20th November 2017).
9. Public Health England. Seasonal influenza vaccine uptake in children of primary school age: winter season 2016 to 2017. Final data for 1 September 2016 to 31 January 2017. Accessed on 17th August 2017 at: https://www.gov.uk/government/uploads/system/uploads/attachment_data/file/613457/Seasonal_influenza_vaccine_uptake_in_children_of_primary_school_age_2016_to_2017.pdf.
10. Rajaram S, Steffey A, Blak B, Hickman M, Christensen H, Caspard H. Uptake of childhood influenza vaccine from 2012-2013 to 2014-2015 in the UK and the implications for high-risk children: a retrospective observational cohort study. BMJ Open. 2016;6(8):e010625.
11. Pebody R, Warburton F, Ellis J, Andrews N, Potts A, Cottrell S, et al. Effectiveness of seasonal influenza vaccine for adults and children in preventing laboratory-confirmed influenza in primary care in the United Kingdom: 2015/16 end-of-season results. Euro Surveill. 2016;21(38):doi: 10.2807/1560-7917.es.2016.21.38.30348.
12. Jefferson T, Jones M, Doshi P, Spencer EA, Onakpoya I, Heneghan CJ. Oseltamivir for influenza in adults and children: systematic review of clinical study reports and summary of regulatory comments. Bmj. 2014;348:g2545.
13. Johnston SL, Ferrero F, Garcia ML, Dutkowski R. Oral oseltamivir improves pulmonary function and reduces exacerbation frequency for influenza-infected children with asthma. Pediatr Infect Dis J. 2005;24:225-32.
14. NICE. National Institute for Health and Clinical Excellence (NICE). Respiratory tract infections - antibiotic prescribing. NICE Clinical Guideline 69. London: Centre for Clinical Practice at NICE, 2008. 2008.
15. Maeda S, Yamada Y, Nakamura H, Maeda T. Efficacy of antibiotics against influenza-like illness in an influenza epidemic. Paediatr Int. 1999;41:274-6.
16. Harnden A, Perera R, Brueggemann AB, Mayon-White R, Crook DW, Thomson A, et al. Respiratory infections for which general practitioners consider prescribing an antibiotic: a prospective study. Arch Dis Child. 2007;92:594-7.
17. Klein EY, Monteforte B, Gupta A, Jiang W, May L, Hsieh YH, et al. The frequency of influenza and bacterial coinfection: a systematic review and meta-analysis. Influenza Other Respir Viruses. 2016;10(5):394-403.
18. McDanel JS, Perencevich EN, Storm J, Diekema DJ, Herwaldt L, Johnson JK, et al. Increased Mortality Rates Associated with Staphylococcus aureus and Influenza Co-infection, Maryland and Iowa, USA(1). Emerg Infect Dis. 2016;22(7):1253-6.
19. Wong KK, Jain S, Blanton L, Dhara R, Brammer L, Fry AM, et al. Influenza-associated pediatric deaths in the United States, 2004-2012. Pediatrics. 2013;132(5):796-804.
20. Goossens H, Ferech M, Vander Stichele R, Elseviers M. Outpatient antibiotic use in Europe and association with resistance: a cross-national database study. Lancet. 2005;365(9459):579-87.
21. National Institute for Health and Care Excellence (NICE). Antimicrobial stewardship: changing risk-related behaviours in the general population. NICE guideline [NG63] Published date: January 2017. https://www.nice.org.uk/guidance/ng63 (Accessed 20th November 2017).
22. Little P, Stuart B, Moore M, Coenen S, Butler CC, Godycki-Cwirko M, et al. Amoxicillin for acute lower respiratory tract infection in primary care when pneumonia is not suspected: a 12-country, randomised, placebo-controlled trial. Lancet Infect Dis. 2013;2013(13):123-9.
23. Meier CR, Napalkov PN, Wegmuller Y, Jefferson T, Jick H. Population-based study on incidence, risk factors, clinical complications and drug utilisation associated with influenza in the United Kingdom. Eur J Clin Microbiol Infect Dis. 2000;19:834-42.
24. Stott NCH. Management and outcome of winter upper respiratory tract infections in children aged 0-9 years. BMJ. 1979;1:29-31.

For peer review only - http://bmjopen.bmj.com/site/about/guidelines.xhtml

25. Adams G, Gulliford MC, Ukoumunne OC, Eldridge S, Chinn S, Campbell MJ. Patterns of intra-cluster correlation from primary care research to inform study design and analysis. J Clin Epidemiol. 2004;57:785-94.
26. Woodcock AJ, Kinmonth AL, Campbell MJ, Griffin SJ, Spiegal NM. Diabetes care from diagnosis: effects of training in patient-centred care on beliefs, attitudes and behaviour of primary care professionals. Patient Educ Couns. 1999;37:65-79.
27. Eldridge SM, Ashby D, Kerry S. Sample size for cluster randomized trials: the effect of coefficient of variation of cluster size and analysis method. Int J Epidemiol. 2006;35:1292-300.
28. Schulz KF, Altman DG, Moher D. CONSORT 2010 statement: updated guidelines for reporting parallel group randomised trials. Bmj. 2010;340:c332.
29. GlaxoSmithKline. Augmentin-Duo 400/57 - Summary of Product Characteristics. Accessed at http://www.medicines.org.uk/emcmobile/medicine/2026/spc on 25/03/2013. 2012.
30. Ashdown HF, Raisanen U, Wang K, Ziebland S, Harnden A. Prescribing antibiotics to 'at-risk' children with influenza-like illness in primary care: qualitative study. BMJ Open. 2016;6(6):e011497.
31. Chung A, Perera R, Brueggemann AB, Elamin AE, Harnden A, Mayon-White R, et al. Effect of antibiotic prescribing on antibiotic resistance in individual children in primary care: prospective cohort study. BMJ. 2007;335:429.
32. van Hecke O, Wang K, Lee JJ, Roberts NW, Butler CC. Implications of Antibiotic Resistance for Patients' Recovery From Common Infections in the Community: A Systematic Review and Meta-analysis. Clin Infect Dis. 2017;65(3):371-82.
33. Public Health England. Surveillance of influenza and other respiratory viruses in the United Kingdom: winter 2014 to 2015. Accessed on 17th August 2017 at: https://www.gov.uk/government/uploads/system/uploads/attachment_data/file/429617/Annualreport_March2015_ver4.pdf.
34. Public Health England. Surveillance of influenza and other respiratory viruses in the United Kingdom: Winter 2015 to 2016. Accessed on 17th August 2017 at: https://www.gov.uk/government/uploads/system/uploads/attachment_data/file/526405/Flu_Annual_Report_2015_2016.pdf.
35. Public Health England. Surveillance of influenza and other respiratory viruses in the UK: Winter 2016 to 2017. Accessed on 17th August 2017 at: https://www.gov.uk/government/uploads/system/uploads/attachment_data/file/613493/Surveillance_of_influenza_and_other_respiratory_viruses_in_the_UK_2016_to_2017.pdf.
36. Francis NA, Ridd MJ, Thomas-Jones E, Butler CC, Hood K, Shepherd V, et al. Oral and Topical Antibiotics for Clinically Infected Eczema in Children: A Pragmatic Randomized Controlled Trial in Ambulatory Care. Ann Fam Med. 2017;15(2):124-30.

Acknowledgements: We would like to thank the ARCHIE Programme Investigators and ARCHIE Programme Steering Committee for their support with producing this protocol. We would also like to thank patient representatives from the NIHR Medicines for Children Research Network Clinical Study Groups, the parents and young people who took part in our discussion groups, and our patient advisers, Farrah Pradhan and Robert Newby, for their input with study design and strategies for delivery.

Authors' contributions: Kay Wang, Tricia Carver and Sharon Tonner drafted the manuscript. Kay Wang is Chief Investigator of the ARCHIE trial. Tricia Carver is the Senior Trial Manager and Sharon Tonner is the Trial Manager. Malcolm G Semple, Alastair Hay, Michael Moore, Paul Little, Christopher Butler, Andrew Farmer and Anthony Harnden contributed to development of the trial protocol and planning of recruitment strategies. Malcolm G Semple also provided input with aspects of the trial relating to microbiological assessments. Malcolm G Semple is the regional Principal Investigator (PI) of the trial in Liverpool, Alastair Hay is regional PI in Bristol, and Michael Moore is

For peer review only - http://bmjopen.bmj.com/site/about/guidelines.xhtml

regional PI in Southampton. Anthony Harnden is the ARCHIE programme lead. Ly-Mee Yu, Susan Mallett and Rafael Perera provided input with statistical aspects of the trial and developed the plan for statistical analysis of trial data. Jane Wolstenholme developed the economic evaluation plan and is leading the economic evaluation of trial findings. All authors have reviewed and contributed edits to the manuscript.

Funding statement: The ARCHIE trial is funded by the National Institute for Health Research (NIHR) under its Programme Grants for Applied Research Programme (Grant Reference RP-PG-1210-12012). The views expressed are those of the authors and not necessarily those of the UK National Health Service, the NIHR, or the UK Department of Health.

Competing interests statement: Kay Wang is a NIHR Postdoctoral Fellow. Andrew Farmer is a NIHR Senior Investigator and is supported by the Oxford NIHR Biomedical Research Centre. Anthony Harnden is Deputy Chairman of the Joint Committee on Vaccination and Immunisation, which advises the UK government on vaccine policy. Malcolm G Semple is a NIHR Investigator, is supported by the NIHR Health Protection Research Unit in Emerging and Zoonotic Infections and is a member of the New and Emerging Respiratory Viral Threats Advisory Group (NERVTAG), whose remit includes advice to the UK government on antibiotic and antiviral stockpile policy.

For peer review only

For peer review only - http://bmjopen.bmj.com/site/about/guidelines.xhtml

Appendix A: Laboratory analysis of throat swabs

Where possible, throat swabs will be obtained from participants at baseline for culture and sensitivity, and in a sub group at 3, 6 and 12 months. The swab will have a broth medium to improve retrieval of the target organisms, which are *Staphylococcus aureus*, *Streptococcus pneumoniae* and *Haemophilus influenzae*. Aliquots of the broth will be pipetted onto selective agar for each of the different organisms. A fourth plate, a selective agar for *Haemophilus influenzae* with ampicillin in the medium at 2mg/L will also be inoculated. Identification of target organisms will be performed in line with Public Health England Standards for Microbiological Investigation methods and susceptibility testing performed in accordance with the latest British Society of Antimicrobial Chemotherapy (BSAC) guidelines. The table below summarises the antimicrobials that will be used for susceptibility testing. Residual broth and isolates of the target organisms will then be put into long-term storage for potential further molecular analysis of resistance and determination of phenotypic resistance. The broth will be frozen to either -70°C or -80°C, in effect snap frozen. Thus, there will be no preservation of intact cells. Assessment of minimum inhibitory concentrations (MICs) will be performed using the agar stipulated in BSAC guidelines.

**Antimicrobial**	**Method**	***S.aureus***	***S.pneumoniae***	***H.influenzae***
Penicillin	MIC	No	Yes	No
Amoxicillin	MIC	No	No	Yes
Co-amoxiclav	MIC	Yes	Yes	Yes
Cefoxitin	MIC	Yes	No	No
Cefotaxime	MIC	No	Yes	Yes
Moxifloxacin	MIC	Yes	Yes	Yes
Erythromycin	MIC	Yes	Yes	No
Nalidixic acid	Disc 30µg	No	No	Yes

Table footnote:

MIC = Minimum Inhibitory Concentration
S. aureus = Staphylococcus aureus
S. pneumoniae = Streptococcus pneumoniae
H. influenzae = Haemophilus influenzae

For peer review only

Appendix B: Data collection

Procedures	Enrolment		Post allocation (trial)			Close out
	V1	V2	Diary	T1	T2	NR
Eligibility assessment	x					
Informed consent +/- assent		x				
Baseline assessment		x				
Nasal swab		x				
Throat swab*		x				
Randomisation		x				
Dispensing of study drug		x				
Allocation of study diary and pack		x				
Adverse events assessment				x	x	
Medical notes review						x
**Assessments**						
Age		x				
Sex		x				
Co-morbidity		x				x
Household smoking status		x				
Vaccination status		x				x
Antivirals/other medications		x				x
Regular medications						x
Heart rate		x				
Respiratory rate		x				
Baseline annual consultation rate						x
Re-consultations due to clinical deterioration						x
Duration of fever		x	x	x	x	
Duration of symptoms		x	x	x	x	
Further medications and/or further investigations						x
Adverse events				x	x	x
Hospitalisations/death				x	x	x
Health care resource use			x			x
EQ-5D-Y proxy		x	x			

V1 = Visit 1; V2 = Visit 2 (conducted within 24 hours of visit 1); T1 = telephone follow-up 1 (one week after randomisation); T2 = telephone follow-up 2 (two weeks after randomisation); NR = Notes Review (i.e. review of participant's medical record).

For peer review only

STANDARD PROTOCOL ITEMS: RECOMMENDATIONS FOR INTERVENTIONAL TRIALS

SPIRIT 2013 Checklist: Recommended items to address in a clinical trial protocol and related documents*

Section/item	Item No	Description	Addressed on page number
**Administrative information**			
Title	1	Descriptive title identifying the study design, population, interventions, and, if applicable, trial acronym	Protocol: 1<br>Manuscript: 1
Trial registration	2a	Trial identifier and registry name. If not yet registered, name of intended registry	Protocol:1<br>Manuscript: 2
	2b	All items from the World Health Organization Trial Registration Data Set	Protocol: 7,8,9
Protocol version	3	Date and version identifier	Protocol: 1
Funding	4	Sources and types of financial, material, and other support	Protocol: 37<br>Manuscript: 18
Roles and responsibilities	5a	Names, affiliations, and roles of protocol contributors	Protocol: 1,2<br>Manuscript: 1
	5b	Name and contact information for the trial sponsor	Protocol: 5
	5c	Role of study sponsor and funders, if any, in study design; collection, management, analysis, and interpretation of data; writing of the report; and the decision to submit the report for publication, including whether they will have ultimate authority over any of these activities	Protocol:37,38<br>Manuscript: 18

For peer review only

For peer review only - http://bmjopen.bmj.com/site/about/guidelines.xhtml

	5d	Composition, roles, and responsibilities of the coordinating centre, steering committee, endpoint adjudication committee, data management team, and other individuals or groups overseeing the trial, if applicable (see Item 21a for data monitoring committee)	Protocol: 5,6,33,35,36
**Introduction**			
Background and rationale	6a	Description of research question and justification for undertaking the trial, including summary of relevant studies (published and unpublished) examining benefits and harms for each intervention	Protocol: 11-14<br>Manuscript: 4-5
	6b	Explanation for choice of comparators	Protocol: 13<br>Manuscript: 6
Objectives	7	Specific objectives or hypotheses	Protocol: 14-16<br>Manuscript: 5
Trial design	8	Description of trial design including type of trial (eg, parallel group, crossover, factorial, single group), allocation ratio, and framework (eg, superiority, equivalence, noninferiority, exploratory)	Protocol: 16<br>Manuscript: 6
**Methods: Participants, interventions, and outcomes**			
Study setting	9	Description of study settings (eg, community clinic, academic hospital) and list of countries where data will be collected. Reference to where list of study sites can be obtained	Protocol: 19<br>Manuscript: 8
Eligibility criteria	10	Inclusion and exclusion criteria for participants. If applicable, eligibility criteria for study centres and individuals who will perform the interventions (eg, surgeons, psychotherapists)	Protocol: 17-19<br>Manuscript: 6-8
Interventions	11a	Interventions for each group with sufficient detail to allow replication, including how and when they will be administered	Protocol: 25<br>Manuscript:9
	11b	Criteria for discontinuing or modifying allocated interventions for a given trial participant (eg, drug dose change in response to harms, participant request, or improving/worsening disease)	Protocol: 24<br>Manuscript: 13

For peer review only - http://bmjopen.bmj.com/site/about/guidelines.xhtml

	11c	Strategies to improve adherence to intervention protocols, and any procedures for monitoring adherence (eg, drug tablet return, laboratory tests)	Protocol: 22-24,35<br>Manuscript: 10
	11d	Relevant concomitant care and interventions that are permitted or prohibited during the trial	Protocol: 26,27<br>Manuscript: 9
Outcomes	12	Primary, secondary, and other outcomes, including the specific measurement variable (eg, systolic blood pressure), analysis metric (eg, change from baseline, final value, time to event), method of aggregation (eg, median, proportion), and time point for each outcome. Explanation of the clinical relevance of chosen efficacy and harm outcomes is strongly recommended	Protocol: 14-16<br>Manuscript: 10
Participant timeline	13	Time schedule of enrolment, interventions (including any run-ins and washouts), assessments, and visits for participants. A schematic diagram is highly recommended (see Figure)	Protocol: 43<br>Manuscript: Appendix B
Sample size	14	Estimated number of participants needed to achieve study objectives and how it was determined, including clinical and statistical assumptions supporting any sample size calculations	Protocol: 33<br>Manuscript: 11-12
Recruitment	15	Strategies for achieving adequate participant enrolment to reach target sample size	Protocol: 19,20<br>Manuscript: 8,15

Methods: Assignment of interventions (for controlled trials)

Allocation:

Sequence generation	16a	Method of generating the allocation sequence (eg, computer-generated random numbers), and list of any factors for stratification. To reduce predictability of a random sequence, details of any planned restriction (eg, blocking) should be provided in a separate document that is unavailable to those who enrol participants or assign interventions	Protocol: 20,21<br>Manuscript: 11
Allocation concealment mechanism	16b	Mechanism of implementing the allocation sequence (eg, central telephone; sequentially numbered, opaque, sealed envelopes), describing any steps to conceal the sequence until interventions are assigned	Protocol: 20,21<br>Manuscript: 11
Implementation	16c	Who will generate the allocation sequence, who will enrol participants, and who will assign participants to interventions	Protocol: 20,21<br>Manuscript: 11

For peer review only - http://bmjopen.bmj.com/site/about/guidelines.xhtml

Blinding (masking)	17a	Who will be blinded after assignment to interventions (eg, trial participants, care providers, outcome assessors, data analysts), and how	Protocol: 21, 26<br>Manuscript: 11
	17b	If blinded, circumstances under which unblinding is permissible, and procedure for revealing a participant's allocated intervention during the trial	Protocol: 21<br>Manuscript: 11
**Methods: Data collection, management, and analysis**			
Data collection methods	18a	Plans for assessment and collection of outcome, baseline, and other trial data, including any related processes to promote data quality (eg, duplicate measurements, training of assessors) and a description of study instruments (eg, questionnaires, laboratory tests) along with their reliability and validity, if known. Reference to where data collection forms can be found, if not in the protocol	Protocol: 21-24<br>Manuscript: 10-11
	18b	Plans to promote participant retention and complete follow-up, including list of any outcome data to be collected for participants who discontinue or deviate from intervention protocols	Protocol: 23,24<br>Manuscript: 13
Data management	19	Plans for data entry, coding, security, and storage, including any related processes to promote data quality (eg, double data entry; range checks for data values). Reference to where details of data management procedures can be found, if not in the protocol	Protocol: 34
Statistical methods	20a	Statistical methods for analysing primary and secondary outcomes. Reference to where other details of the statistical analysis plan can be found, if not in the protocol	Protocol: 30-32<br>Manuscript: 12
	20b	Methods for any additional analyses (eg, subgroup and adjusted analyses)	Protocol: 30-32<br>Manuscript: 12
	20c	Definition of analysis population relating to protocol non-adherence (eg, as randomised analysis), and any statistical methods to handle missing data (eg, multiple imputation)	Protocol: 33<br>Manuscript: 12
**Methods: Monitoring**			
Data monitoring	21a	Composition of data monitoring committee (DMC); summary of its role and reporting structure; statement of whether it is independent from the sponsor and competing interests; and reference to where further details about its charter can be found, if not in the protocol. Alternatively, an explanation of why a DMC is not needed	Protocol: 6,7,30,36<br>Manuscript: 13

For peer review only - http://bmjopen.bmj.com/site/about/guidelines.xhtml

	21b	Description of any interim analyses and stopping guidelines, including who will have access to these interim results and make the final decision to terminate the trial	Protocol: 33<br>Manuscript: 13
Harms	22	Plans for collecting, assessing, reporting, and managing solicited and spontaneously reported adverse events and other unintended effects of trial interventions or trial conduct	Protocol: 27-30<br>Manuscript: 12-13
Auditing	23	Frequency and procedures for auditing trial conduct, if any, and whether the process will be independent from investigators and the sponsor	Protocol: 35
**Ethics and dissemination**			
Research ethics approval	24	Plans for seeking research ethics committee/institutional review board (REC/IRB) approval	Protocol: 36<br>Manuscript: 15
Protocol amendments	25	Plans for communicating important protocol modifications (eg, changes to eligibility criteria, outcomes, analyses) to relevant parties (eg, investigators, REC/IRBs, trial participants, trial registries, journals, regulators)	Protocol: 36,45<br>Manuscript: 15-16
Consent or assent	26a	Who will obtain informed consent or assent from potential trial participants or authorised surrogates, and how (see Item 32)	Protocol: 20<br>Manuscript: 8
	26b	Additional consent provisions for collection and use of participant data and biological specimens in ancillary studies, if applicable	Protocol: 20<br>Manuscript:10
Confidentiality	27	How personal information about potential and enrolled participants will be collected, shared, and maintained in order to protect confidentiality before, during, and after the trial	Protocol: 37<br>Manuscript: 16
Declaration of interests	28	Financial and other competing interests for principal investigators for the overall trial and each study site	Manuscript: 18
Access to data	29	Statement of who will have access to the final trial dataset, and disclosure of contractual agreements that limit such access for investigators	Protocol: 34
Ancillary and post-trial care	30	Provisions, if any, for ancillary and post-trial care, and for compensation to those who suffer harm from trial participation	Protocol: 37

For peer review only

For peer review only - http://bmjopen.bmj.com/site/about/guidelines.xhtml

Dissemination policy	31a	Plans for investigators and sponsor to communicate trial results to participants, healthcare professionals, the public, and other relevant groups (eg, via publication, reporting in results databases, or other data sharing arrangements), including any publication restrictions	Manuscript: 15-16
	31b	Authorship eligibility guidelines and any intended use of professional writers	Protocol: 38
	31c	Plans, if any, for granting public access to the full protocol, participant-level dataset, and statistical code	_not applicable_
**Appendices**			
Informed consent materials	32	Model consent form and other related documentation given to participants and authorised surrogates	Additional appendix
Biological specimens	33	Plans for collection, laboratory evaluation, and storage of biological specimens for genetic or molecular analysis in the current trial and for future use in ancillary studies, if applicable	Protocol: 22 Manuscript: 10

*It is strongly recommended that this checklist be read in conjunction with the SPIRIT 2013 Explanation & Elaboration for important clarification on the items. Amendments to the protocol should be tracked and dated. The SPIRIT checklist is copyrighted by the SPIRIT Group under the Creative Commons "Attribution-NonCommercial-NoDerivs 3.0 Unported" license.

For peer review only

For peer review only - http://bmjopen.bmj.com/site/about/guidelines.xhtml
